# Hydrogels and Nanogels: Pioneering the Future of Advanced Drug Delivery Systems

**DOI:** 10.3390/pharmaceutics17020215

**Published:** 2025-02-07

**Authors:** Ernesto J. Delgado-Pujol, Guillermo Martínez, David Casado-Jurado, Juan Vázquez, Jesús León-Barberena, David Rodríguez-Lucena, Yadir Torres, Ana Alcudia, Belén Begines

**Affiliations:** 1 Departamento de Química Orgánica y Farmacéutica, Facultad de Farmacia, Universidad de Sevilla, 41012 Sevilla, Spain; edelgado4@us.es (E.J.D.-P.); gmartinez1@us.es (G.M.); davcasjur@alum.us.es (D.C.-J.); drodriguez5@us.es (D.R.-L.); aalcudia@us.es (A.A.); 2 Departamento de Ingeniería y Ciencia de los Materiales y del Transporte, Escuela Politécnica Superior, Universidad de Sevilla, 41011 Sevilla, Spain; ytorres@us.es; 3 Departamento de Citología e Histología Normal y Patológica, Facultad de Medicina, Universidad de Sevilla, 41009 Sevilla, Spain; 4 Departamento de Química Orgánica, Facultad de Química, Universidad de Sevilla, 41012 Sevilla, Spain; jesleobar@alum.us.es

**Keywords:** hydrogels for drug delivery, nanogels in therapeutics, stimuli-responsive drug delivery systems, personalized medicine with hydrogels, biocompatible polymer networks

## Abstract

Conventional drug delivery approaches, including tablets and capsules, often suffer from reduced therapeutic effectiveness, largely attributed to inadequate bioavailability and difficulties in ensuring patient adherence. These challenges have driven the development of advanced drug delivery systems (DDS), with hydrogels and especially nanogels emerging as promising materials to overcome these limitations. Hydrogels, with their biocompatibility, high water content, and stimuli-responsive properties, provide controlled and targeted drug release. This review explores the evolution, properties, and classifications of hydrogels versus nanogels and their applications in drug delivery, detailing synthesis methods, including chemical crosslinking, physical self-assembly, and advanced techniques such as microfluidics and 3D printing. It also examines drug-loading mechanisms (e.g., physical encapsulation and electrostatic interactions) and release strategies (e.g., diffusion, stimuli-responsive, and enzyme-triggered). These gels demonstrate significant advantages in addressing the limitations of traditional DDS, offering improved drug stability, sustained release, and high specificity. Their adaptability extends to various routes of administration, including topical, oral, and injectable forms, while emerging nanogels further enhance therapeutic targeting through nanoscale precision and stimuli responsiveness. Although hydrogels and nanogels have transformative potential in personalized medicine, challenges remain in scalable manufacturing, regulatory approval, and targeted delivery. Future strategies include integrating biosensors for real-time monitoring, developing dual-stimuli-responsive systems, and optimizing surface functionalization for specificity. These advancements aim to establish hydrogels and nanogels as cornerstones of next-generation therapeutic solutions, revolutionizing drug delivery, and paving the way for innovative, patient-centered treatments.

## 1. Introduction

Traditional drug delivery methods, including tablets, capsules, and syrups, have long been the cornerstone of pharmacological treatments. However, these conventional approaches present significant limitations in therapeutic efficacy, including challenges with drug stability and patient compliance. A primary limitation of these methods is their low bioavailability, defined as the proportion of the drug that successfully reaches the systemic circulation to exert its therapeutic effect. This inefficiency can be attributed to physiological barriers, such as degradation within the gastrointestinal tract and hepatic first-pass metabolism, which considerably reduce the effective drug reaching the target site [1]. Additionally, conventional delivery often results in rapid drug release, causing an initial spike in plasma concentration followed by a rapid decline. This fluctuation requires frequent dosing to maintain therapeutic levels, which can increase the risk of side effects and reduce patient adherence [2].

To address these challenges, the field of drug delivery has shifted towards advanced drug delivery systems (DDS), which offer innovative strategies to enhance therapeutic efficacy by controlling the dosage, timing, and site of drug release [3]. Among various DDS technologies, hydrogels have emerged as one of the most promising materials due to their unique physical and chemical properties, enabling the controlled release of therapeutic agents and targeted delivery to specific tissues. Hydrogels are highly hydrated three-dimensional polymer networks that are capable of encapsulating and releasing drugs over a sustained period, making them suitable for treatments that require prolonged or targeted release [4,5]. Hydrogels were introduced in the early 1960s and pioneered by Wichterle and D. Lim [6], who developed the first hydrogel, poly (2-hydroxyethyl methacrylate) for use in contact lenses. This discovery marked the beginning of modern hydrogel technology, inspiring decades of research into hydrogels for biomedical applications beyond ocular use [7]. Since then, hydrogels have evolved significantly, with research expanding their applications to controlled drug delivery, where they have become essential due to their biocompatibility, high water content, and ability to mimic natural tissues [8]. These characteristics allow hydrogels to encapsulate drugs while protecting them from enzymatic degradation, particularly important for sensitive biomolecules like peptides and proteins. Modern hydrogel systems are often described as “smart” materials due to their ability to respond to physiological stimuli such as pH, temperature, or biochemical signals, enabling precise, on-demand drug release [9]. This responsiveness allows smart hydrogels to release therapeutic agents selectively in target environments, such as acidic tumor tissues or inflamed regions, where specific conditions trigger drug release. This adaptability is crucial for applications requiring high specificity, like cancer treatment, where minimizing systemic toxicity is paramount [10].

**Figure 1:**
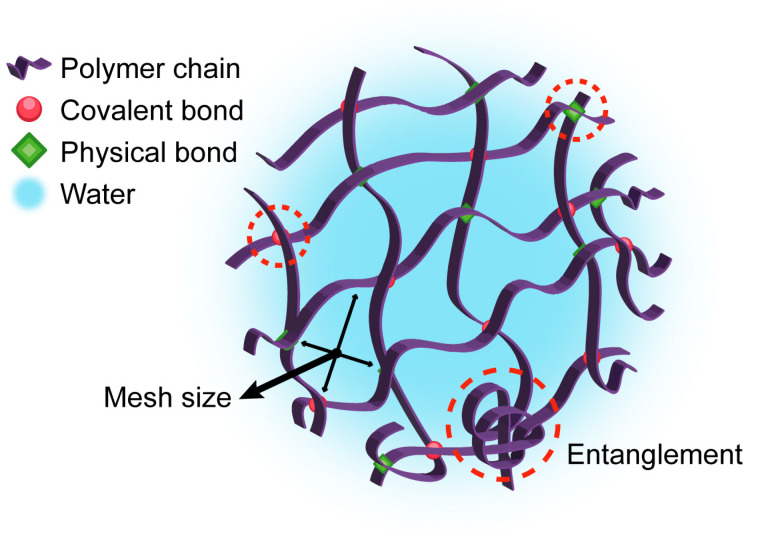
General structure of a hydrogel, including the potential polymer interactions required to create and reinforce the 3D structure.

In terms of structure, hydrogels are formed by crosslinked polymer chains, arranged in a three-dimensional network that stabilizes the material. This crosslinked structure enables the hydrogel to retain a practically ‘infinite’ molecular weight, a concept that, while not literal, highlights the unique network properties not observed in simpler polymers [11]. Figure 1 illustrates the general structure of a hydrogel, highlighting key features such as the crosslinked polymer network and possible bonding types, including both covalent and reversible interactions that contribute to the stability and responsiveness of the hydrogel [12]. The 3D configuration of the material is produced by the crosslinking of different polymer chains that appear, in turn, through the generation of chemical bonds (permanent connections that provide stability of the network) or physical bonds (interactions such as hydrogen bonds or hydrophobic interactions that can break and reform, allowing some flexibility and response to stimulus in the hydrogel). The appearance of entanglement is also common due to the physical interconnection of polymer chains with each other without forming chemical bonds [12]. Although covalent bonds provide a more stable and robust structure, physical bonds, although weaker, allow for the reversibility and flexibility of the hydrogel. This duality in crosslinking methods broadens the range of possible applications for hydrogels [13].

The versatility of hydrogels allows them to be administered through various routes, each offering unique advantages depending on therapeutic requirements and target tissue:

1. Topical Application: Hydrogels are ideal for topical use because of their high water content, which provides a moist environment beneficial for wound healing and tissue repair. They are widely applied in skin treatments, burns, and ulcers. Nanogels, with their enhanced penetration abilities, can deliver drugs deeper into the skin, making them effective for localized cancer therapies and the treatment of chronic skin diseases [14].
2. Oral Administration: Oral hydrogels are designed to swell upon contact with gastrointestinal fluids, gradually releasing drugs as they move through the GI tract. Bioadhesive hydrogels adhere to the mucosal surface, enhancing bioavailability for drugs with poor absorption rates. This sustained release can be beneficial for drugs that require prolonged delivery or protection against gastric degradation [15].
3. Buccal Administration: Due to their bioadhesive properties, hydrogels are suitable for oral drug delivery, allowing sustained release through the buccal mucosa and bypassing first-pass metabolism, which is advantageous for drugs susceptible to liver degradation [16].
4. Injectable Administration: Injectable hydrogels offer a minimally invasive option for sustained drug release directly into the systemic circulation or targeted tissues. After injection, these hydrogels form stable depots that gradually release the drug, improving therapeutic outcomes by focusing the treatment locally. Injectable nanogels add additional targeting capabilities, minimizing systemic side effects [17].
5. Other Routes: Hydrogels are also utilized in transdermal patches, ophthalmic solutions, and intranasal delivery systems. For instance, intranasal nanogels bypass the blood–brain barrier, enabling direct delivery of therapeutics to the central nervous system [18].

Despite their promise, hydrogels face several technical challenges that limit their widespread clinical use. Issues such as precise control over gelation, potential immunogenicity, and challenges in scaling up production for clinical applications remain areas of active research [19]. Researchers continue to explore ways to improve the performance of hydrogels, focusing on new crosslinking strategies, enhanced stability, and the development of hydrogels that can degrade safely in the body after treatment [20]. The integration of nanocarriers, such as nanoparticles or liposomes, into hydrogel matrices also represents a promising strategy to enhance drug loading capacity and release profiles, broadening the therapeutic potential of these systems [21].

**Figure 2:**
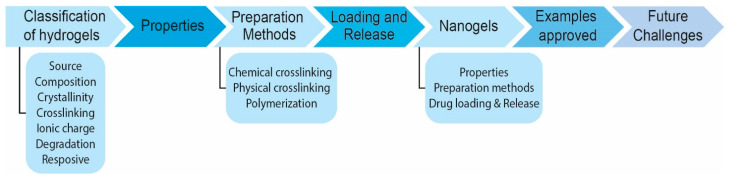
Schematics of the different sections addressed in this literature review.

In this review, different aspects of hydrogels are explored, including their physicochemical properties, their classifications, and the various types of hydrogels and nanogels used in drug delivery applications (Figure 2). The challenges and recent innovations in hydrogel technology are also discussed, emphasizing the role of hydrogels in advancing personalized medicine and sustainable therapeutic solutions. Through these explorations, the aim was to highlight the transformative potential of hydrogel-based DDS to overcome the limitations of conventional drug delivery methods and to pave the way for new, patient-centered therapeutic strategies.

## 2. Classification of Hydrogels

**Figure 3:**
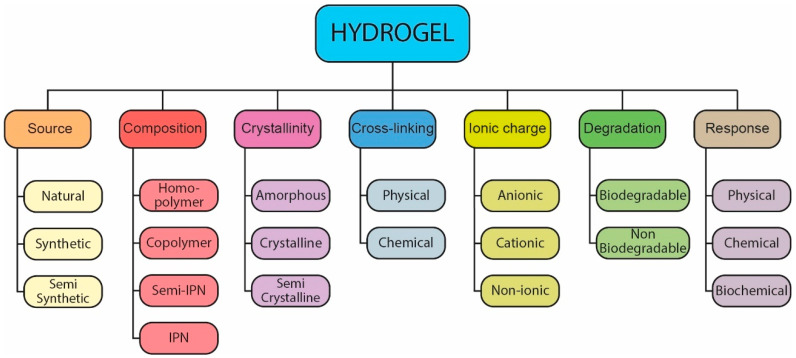
Classification of hydrogels.

Hydrogels can be classified on the basis of various criteria, including their source, composition, crosslinking methods, and responsiveness to environmental stimuli. This classification not only provides a framework for understanding the structural and functional diversity of hydrogels but also serves as a guide for selecting appropriate hydrogels for specific drug delivery applications (see Figure 3 for a schematic overview of the hydrogel classification).

Source: Hydrogels are derived from natural or synthetic polymers. Natural hydrogels, such as those based on chitosan, collagen, or hyaluronic acid, are known for their biocompatibility, biodegradability, and intrinsic bioactivity, making them ideal for applications requiring high biological integration. However, they may lack mechanical strength and sometimes require reinforcement [22]. Synthetic hydrogels, made from polymers like poly(ethylene glycol) (PEG) and poly(vinyl alcohol) (PVA), offer greater control over mechanical and physicochemical properties, although they may require surface modifications to improve biocompatibility and reduce immunogenicity [12]. Semi-synthetic hydrogels combine natural and synthetic polymers to optimize both biological activity and mechanical properties, such as acrylate-modified hyaluronic acid [23].

**Figure 4:**
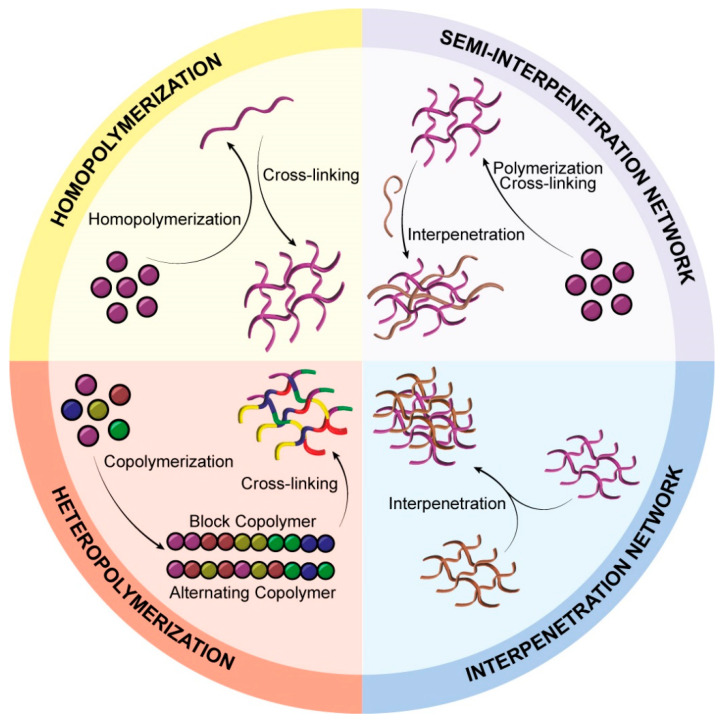
Classification of hydrogels according to their composition.

Composition: Hydrogels can be composed of a single type of monomer (homopolymers) or multiple types (copolymers), where at least one hydrophilic monomer facilitates water absorption (Figure 4). Interpenetrating polymer networks (IPNs) and semi-IPNs are advanced forms of hydrogel structures. IPNs consist of two crosslinked polymers interwoven within each other, while semi-IPNs integrate a crosslinked polymer network with a non-crosslinked counterpart, providing a combination of stability and flexibility ideal for drug delivery applications where adaptability and structural integrity are essential [24].

Crystallinity: According to the crystallization state of the polymer, hydrogels can be found as amorphous (an irregular random polymeric network with no crystalline order); crystalline (a highly compact and ordered polymer network) or semicrystalline (contains both amorphous and crystalline regions, with rapid phase transitions useful for injectable hydrogels and shape memory applications) [12,23].

Crosslinking Method: Crosslinking, a critical aspect of the hydrogel structure, affects the stability and swelling behavior. Chemically crosslinked hydrogels, formed through covalent bonds, provide stable structures that are less susceptible to environmental changes, which can be beneficial for long-term drug release applications. In contrast, physically crosslinked hydrogels, which rely on ionic or hydrogen bonds, offer greater flexibility and responsiveness, allowing the hydrogel to adapt to stimuli in the physiological environment [25].

Ionic charge: Hydrogels can be non-ionic (neutral), ionic (anionic or cationic), amphoteric (containing acidic and basic groups), or zwitterionic (containing both anionic and cationic groups in each repeating unit) [26].

Degradability: Hydrogels can be either durable (e.g., polyacrylate-based) or biodegradable (e.g., polysaccharide-based) [27].

**Figure 5:**
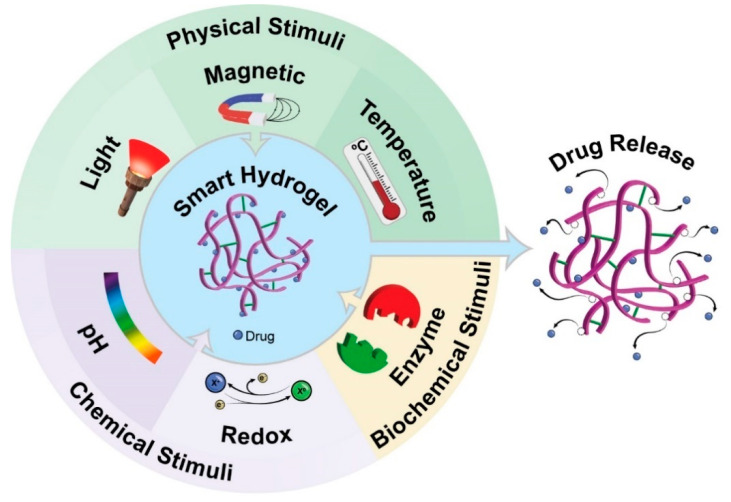
Main stimuli to which a hydrogel can be designed to respond.

Stimuli Responsiveness: Conventional hydrogels undergo slight modifications in their structure, mainly through swelling, in response to external environmental conditions, and have low mechanical strength [23]. However, smart hydrogels are sensitive to small changes in environmental conditions and rapidly adjust their physical properties, such as mechanical strength, swelling capacity, permeability, and stimulus sensitivity [12]. In this sense, smart hydrogels can respond to physical stimuli (such as temperature, pressure, light, or electric and magnetic fields), chemical stimuli (such as pH, ionic strength, solvent composition, or molecular species), or biochemical stimuli (such as antigen, ligand, or enzyme response), affecting properties such as swelling and deformation [23] (Figure 5). For example, pH-sensitive hydrogels are designed to swell in acidic or basic environments, making them suitable for targeting specific regions of the gastrointestinal tract or tumor tissues, where pH variations can be exploited for localized drug release [28]. Similarly, temperature-sensitive hydrogels respond to changes in temperature, expanding or contracting to control drug release as needed [9].

These classification criteria allow for the tailored design of hydrogels to meet specific drug delivery requirements, from sustained release in chronic conditions to responsive, localized release in targeted therapies.

## 3. Physical and Chemical Properties of Hydrogels

The unique structural characteristics of hydrogels are determined by the polymer composition. The type of polymer used determines key parameters such as the biocompatibility, degradability, and drug loading capacity of the hydrogel [29]. In addition, the polymer composition can also affect surface functionality, allowing the introduction of functional groups on the surface of the hydrogel, enhancing their drug-loading capacity and controlled release. Functional groups such as carboxyl, amino, and hydroxyl enable the conjugation of drugs and other therapeutic agents, improving drug stability within the hydrogel [29]. For biomedical applications, biocompatibility is critical to allow hydrogels to interact with biomolecules such as drugs and cells [30]. Similarly, degradability is also an important factor because hydrogels can gradually degrade within the body, releasing drugs in a controlled manner without causing toxic effects. Degradation can be tailored by modifying the polymer composition and crosslinking density [31]. Crosslinking can be achieved chemically, forming permanent covalent bonds, or physically, creating reversible interactions like hydrogen bonds or ionic interactions. These crosslinked structures endow hydrogels with a degree of flexibility, stability, and responsiveness to various stimuli, such as pH, temperature, or mechanical stress, which can be exploited to control the release of encapsulated drugs [13].

Swelling capacity, one of the most crucial properties for drug release, begins with the absorption of water by the hydrophilic groups in the polymer chains. This absorption process allows the hydrogel network to expand, creating channels that facilitate drug diffusion. The degree of swelling is directly influenced by factors such as polymer concentration and crosslinking density; for example, a higher crosslinking density results in a more compact structure with reduced swelling capacity but enhanced mechanical stability, making it more suitable for applications that require longer-lasting release profiles [32]. Porosity influences the transport of nutrients, waste, and molecules through the hydrogel. Greater porosity facilitates the diffusion of larger molecules, while smaller pores allow for controlled drug release [33]. Elasticity and viscoelasticity are additional critical properties in hydrogels, especially in tissue engineering applications where the material must conform to irregular shapes and withstand physiological stresses. The dual behavior of viscoelasticity enables hydrogels to act as both stable and adaptable carriers, ensuring that they remain intact under physiological conditions while allowing gradual drug release [34]. Transparency is crucial in optical applications like contact lenses, allowing light to pass without significant scattering, and maintaining visual clarity [35].

The ability to fine tune these physicochemical properties is particularly valuable in the design of DDSs tailored to specific therapeutic needs, where drug release can be triggered by external or internal cues.

## 4. General Preparation Methods of Hydrogels

Hydrogels are synthesized using various methods, each offering unique advantages in terms of structure, size, and functionality. The choice of preparation method significantly impacts the physicochemical properties of hydrogels, such as size uniformity, mechanical stability, and responsiveness to stimuli.

Chemical Crosslinking: Chemical crosslinking involves the formation of covalent bonds within polymeric networks, yielding highly stable hydrogels. This method is commonly used to produce hydrogels with precise size and robust mechanical properties [36]. The uniformity of the properties usually leads to good control in drug delivery. Common examples of chemical crosslinking are photo-crosslinking, the use of crosslinking agents, or the application of Click chemistry reactions. In photo-crosslinking, UV or visible light is used to initiate crosslinking reactions in certain polymer systems. For example, PEG and poly(vinyl alcohol) hydrogels can be crosslinked using UV light in the presence of a photoinitiator to create stable networks. This approach enables spatial and temporal control of hydrogel formation, making it particularly useful for creating patterned hydrogels and bioinks for 3D bioprinting [37]. Despite its advantages, photo-crosslinking can be limited by the potential cytotoxicity of photoinitiators and the depth of light penetration. Glutaraldehyde is a commonly used agent to crosslink polysaccharides like alginate or chitosan, creating strong, stable hydrogels for biomedical applications such as wound healing. Another example is epoxy-based crosslinkers, which are often used to form hydrogels for controlled drug delivery. This method ensures long-term structural integrity but requires the use of chemical agents, which can introduce cytotoxicity [38]. Click chemistry is a popular technique in hydrogel chemistry. It involves reactions like the azide-alkyne reaction to form highly efficient and specific covalent bonds [39]. This method is often used for creating functionalized hydrogels that can deliver drugs in a controlled manner. Mauri et al. [40] discussed the use of controlled polymerization techniques for precise chemical crosslinking to ensure reproducibility in hydrogel synthesis. It is worth mentioning chemical crosslinking using high-energy radiation, such as gamma rays or electron beams. It can be used to initiate polymerization and crosslinking in hydrogel synthesis. This method is particularly effective for producing sterile and biocompatible hydrogels. It avoids the need for chemical initiators, which makes it suitable for biomedical applications; however, it requires accessibility to high-energy radiation facilities.

Physical Self-Assembly: Physical self-assembly relies on non-covalent interactions such as hydrogen bonding, hydrophobic interactions, or electrostatic forces to form hydrogels. This method is particularly suitable for sensitive drugs or biomolecules like proteins and nucleic acids, as it avoids harsh chemical conditions and preserves their bioactivity [41,42]. Ravarino et al. [43] described the self-assembly of a fluorine-containing dipeptide to form hydrogels via π-π stacking, hydrogen bonding, and halogen bonds. The hydrogel demonstrated excellent physical properties, making it useful for drug delivery and biomedical applications.

Polymerization: Polymerization techniques can also be divided into emulsion polymerization and photopolymerization. Emulsion polymerization is a widely used technique in which monomers are polymerized within a dispersed phase (usually water) to form hydrogels. The method can produce hydrogels with a controlled size and high drug-loading efficiency, leading to scalable production, high size uniformity, and cost-effectiveness. However, it requires surfactants, which may need to be removed post-synthesis. Romero et al. [44] detailed the preparation of hydrogels using emulsion polymerization of *N*-vinyl caprolactam crosslinked with PEG diacrylate. The resulting hydrogels were thermoresponsive, exhibiting phase transitions near physiological temperature, making them suitable for controlled DDS. The drug release behavior was evaluated using colchicine as a model drug, demonstrating sustained and controlled release profiles. Photopolymerization involves initiating polymerization using light. It allows precise spatial and temporal control over nanogel formation. Typically, UV or visible light is used to activate a photoinitiator, leading to crosslinking of the polymer matrix. It allows high control over the polymerization process, minimal side reactions, and environmental responsiveness; however, it requires specialized equipment and may limit scalability. Y. Wang et al. [45] described the preparation of a semi-interpenetrating polymer networks hydrogel composed of PEG diacrylate and hyaluronic acid. The hydrogel, prepared via in situ UV photopolymerization, incorporated paclitaxel-loaded poly(lactic-co-glycolic acid) nanoparticles. The system demonstrated effective local drug delivery, sustained release for up to 13 days, and significant tumor inhibition without toxic effects in vivo.

Each synthesis process offers unique advantages and challenges. Chemical crosslinking is widely used due to its ability to produce hydrogels with robust mechanical properties and tailored functionalities. However, the use of chemical crosslinkers or initiators often introduces cytotoxicity and necessitates extensive purification to ensure biocompatibility, limiting its direct applicability for biomedical purposes [46]. In contrast, physical self-assembly relies on non-covalent interactions such as hydrogen bonding, ionic interactions, and hydrophobic effects to form hydrogels, making it a safer and more environmentally friendly approach. While this method avoids the need for potentially harmful reagents, the resulting hydrogels often exhibit weaker mechanical properties and sensitivity to environmental conditions, which can limit their stability in physiological applications [47]. Polymerization techniques, including free-radical and controlled polymerization, allow for precise control over hydrogel composition and structure, enabling the incorporation of stimuli-responsive properties. However, these methods often require stringent reaction conditions and may involve toxic reagents, posing challenges for clinical translation [48]. Moving forward, hybrid approaches combining the strengths of these techniques while addressing their limitations—such as green chemistry-based crosslinking or advanced self-assembly methods enhanced by polymerization—may offer more versatile and scalable solutions for hydrogel fabrication.

## 5. Drug Loading and Release Mechanisms

Hydrogels provide multiple mechanisms for drug loading, including physical encapsulation, covalent attachment, and electrostatic interactions [49]. This versatility allows for the inclusion of a wide range of therapeutic molecules, from small-molecule drugs to larger biomolecules like proteins and nucleic acids. Physical encapsulation is commonly used for hydrophilic or even hydrophobic drugs [50]. It is based on the physical entrapment of drugs inside hydrophilic vehicles. This method relies on the swelling and contraction properties of the hydrogels to retain the drug inside. Abdel-Rashid et al. [51] prepared a chitosan-based nanogel containing sorbitan monostearate–sodium deoxycholate nanovesicles loaded with acetazolamide for the delivery of ocular drugs. Electrostatic interactions are ideal for loading negatively or positively charged biomolecules, such as siRNA or DNA, which are often used in gene therapy applications [52]. Tang et al. [53] described the use of a DNA hydrogel linked by electrostatic interactions to antisense oligonucleotides. The hydrogel also carried chemotherapeutics, such as doxorubicin, for combined chemo/gene therapy of multidrug-resistant tumors. Covalent attachment is used when ionic species are required; for example, in theragnostic applications. Carniato et al. [54] prepared chitosan-based hydrogels using Mn(II) chelates as both covalent crosslinkers and contrast agents.

Drug release from hydrogels can occur through various pathways, depending on the design and intended application. The most common release mechanisms include the following.

### 5.1. Diffusion-Controlled Release in Conventional Hydrogels

Conventional hydrogels are defined by their stable, static network structure, which allows them to release drugs primarily through a diffusion-controlled mechanism. These hydrogels are particularly valuable for applications requiring sustained or prolonged drug release, as the gradual migration of drug molecules out of the hydrogel matrix provides a consistent dose over time. This characteristic makes conventional hydrogels highly suitable for managing chronic conditions, where maintaining a stable concentration of the drug in the bloodstream is essential to achieve therapeutic efficacy without frequent re-dosing [55].

A significant application of conventional hydrogels is in topical drug formulations, particularly in wound care and skin conditions. Their high water content provides a moist environment that promotes wound healing and tissue regeneration. This moist environment promotes autolytic debridement, a process that removes dead tissue without external intervention. It not only facilitates the migration of regenerative cells to the wound site, but also reduces pain, providing a soothing effect for the patient [56]. For wounds in constantly moving areas, such as joints, self-healing hydrogels with high elasticity and adhesiveness are essential. These properties allow the hydrogel to recover after deformation and remain adhered to the wound, facilitating continuous healing without the need for frequent dressing changes. Notable examples include polyacrylamide and alginate hydrogels with dynamic bonds that offer up to 80% mechanical recovery in a few hours, which is especially useful for injuries requiring high flexibility and strength [57]. Basu et al. [58] described the preparation of calcium crosslinked nanocellulose hydrogel. These nanofibrillated cellulose hydrogels, crosslinked with calcium, formed a fibrous network that retained moisture well, promoting a favorable environment for tissue regeneration. They were shown to be biocompatible and showed potential for use in advanced wound dressings. Other examples included polysaccharide-based hydrogels, based on the functionalization of hydrogels with polymers such as alginate, hyaluronic acid, and chitosan. They showed not only that they retain moisture but also act as barriers against bacteria, making them useful for hard-to-treat wounds. They are suitable for active dressings that respond to external stimuli [59]. J. Li et al. [60] described an in situ forming hydrogel made from oxidized hydroxyethyl starch and modified carboxymethyl chitosan. It provided excellent water retention, biocompatibility, and self-recoverability properties. It promoted wound healing by maintaining a moist environment and adapting to irregular wound shapes, aiding in faster epithelialization and granulation. W. Liu et al. [61] prepared amphiphilic, oxadiazole group-decorated quaternary ammonium salts-conjugated poly(ε-caprolactone)-PEG-poly(ε-caprolactone) hydrogels to fight methicillin-resistant *Staphylococcus aureus* (MRSA) infections. It promoted skin regeneration without additional drugs or light stimuli, making it effective for Gram-positive and Gram-negative bacteria in wound healing. Developed with cationic properties, the hydrogel obtained from chemically cross-linked trans-1,4-cyclohexanediamine with 1,3-dibromo-2-propanol was inherently antibacterial, effectively targeting *Staphylococcus aureus* and *Escherichia coli*. It promoted rapid wound healing by stopping bleeding, supporting collagen deposition, and encouraging blood vessel formation in infected wounds [62].

However, the high water content of hydrogels also provides effectiveness as carriers for delivering therapeutic agents like antimicrobials, which are gradually released to prevent infection and enhance tissue repair [14]. For instance, hydrogel dressings loaded with antibacterial agents offer a noninvasive means to protect against pathogens, maintain optimal moisture levels, and reduce the frequency of dressing changes. In this sense, H. Chen et al. [63] described the use of an alginate–chitosan–tetracycline hydrogel. This composite hydrogel was prepared from an alginate–chitosan polymer network containing gelatine microspheres loaded with tetracycline hydrochloride, which provided sustained antibacterial effects against *E. coli* and *S. aureus*. It also offered high stability and mechanical strength. But in an attempt to avoid bacterial resistance, some researchers have proposed the application of hydrogels containing nonantibiotic structures. Manna et al. [64] described the preparation of a hydrogel using the peptide pool extracted from a formulated curd composed of a blend of probiotic bacteria such as *Streptococcus thermophilus*, *Lactobacillus casei*, and *Bifidobacterium bifidum*. This hydrogel inhibited biofilm formation and effectively accelerated wound healing. It offered an alternative to antibiotics by harnessing natural peptides with strong antibacterial properties. As an alternative, Fan et al. [65] proposed the use of composites prepared from acrylic acid and *N,N*′-methylene bisacrylamide at different mass ratios enriched with silver and graphene to create a dressing with high antibacterial capacity and water retention, accelerating wound healing. Its structure kept the area moist and helped regenerate the epidermis in animal models. In a similar approach, S. Song et al. [66] prepared a hydrogel containing silver nanoparticles integrated into a polyvinyl alcohol and bacterial cellulose matrix. This combination showed excellent mechanical properties and effective antibacterial activity. It promoted fast wound repair with up to 97.89% healing in mice wound models within 15 days. Alginate–chitosan–ZnO hydrogel provided a breathable, moist, and antibacterial environment suitable for wound healing. It maintained high porosity and swelling capacity, promoting cell migration and wound repair effectively in various bacterial infections [67].

Some other authors have proposed loading hydrogels with healing-promoting agents. An example was the oral mucosa-inspired biomimetic hydrogel. This alginate-based hydrogel mimicked the healing process of oral mucosa, combining growth factors and a sterile-moist microenvironment for rapid and scar-free healing. This approach showed promise in reducing scar formation and controlling inflammation, making it suitable for chronic wounds and cosmetic applications [68]. Thangavel et al. [69] developed a chitosan hydrogel containing silk fibroin and L-proline to improve water retention and promote tissue regeneration, showing high biocompatibility and antioxidant activity. Another example is the use of carboxymethyl chitosan hydrogel enriched with epigallocatechin-3-O-gallate, a powerful antioxidant and anti-inflammatory agent. It enhanced wound healing by providing a moist environment, antibacterial activity, and free radical scavenging. This hydrogel was shown to promote collagen deposition, epidermis regeneration, and upregulate vascular endothelial growth factor, all essential for complete tissue repair in full-thickness skin wounds [70].

A major trend in wound healing is the development of injectable hydrogels that gel directly at the wound site. These liquid hydrogels can be applied directly to deep or irregular wounds, forming a gel that adapts to the shape of the wound and provides complete three-dimensional coverage. This method reduces the need for invasive procedures and is well-suited for chronic wounds requiring constant application [71]. One example of an injectable hydrogel is the hydrophobically modified chitosan and oxidized dextrane-based system, which showed excellent compatibility with dermal cells and a significant ability to stop bleeding and prevent infections in infected wound models in rats [72]. Balitaan et al. [73] described the use of glycol chitosan/dibenzaldehyde-terminated polyethylene glycol hydrogel. This self-healing injectable hydrogel had inherent antibacterial properties, providing a moist and adaptable environment for accelerated healing. The material promoted wound contraction and prevented infections in wound models in mice. Targeting multidrug-resistant *S. aureus*, D. Cai et al. [74] self-assembled benzyl 3β-amino-11-oxo-olean-12-en-30-oate hydrogel. It inhibited pro-inflammatory cytokines and enhanced wound healing by supporting collagen deposition and reducing inflammation in infected wounds. The injectable hydrogel developed by H. Chen et al. [75] from multiarm thiolated polyethylene glycol crosslinked with silver ions provided self-healing, antibacterial, and angiogenic capabilities. The addition of desferrioxamine improved blood vessel formation, making this hydrogel particularly effective in the healing of diabetic wounds that are prone to infections. In vivo studies demonstrated accelerated wound healing due to its ability to combat bacterial infections and support angiogenesis. In a similar approach, W. Zhang et al. [76] prepared an injectable hydrogel composed of carboxymethyl chitosan and oxidized hyaluronic acid loaded with blueberry anthocyanins, known for their antioxidant and anti-inflammatory properties. The hydrogel provided a suitable environment for rapid wound closure, promoting tissue regeneration and reducing inflammation. In a rat skin wound model, this hydrogel accelerated healing by enhancing collagen deposition, up-regulating vascular endothelial growth factor, and promoting a shift in macrophages toward a healing (M2) phenotype.

Some authors have explored the possibility of combining different hydrogels or loadings to improve the performance obtained by using only one hydrogel, or even achieving an objective that could not be reached with any of the current hydrogels. For example, J. Wang et al. [77] prepared dual-layer biomimetic hydrogel for cartilage regeneration. They used a hydrogel obtained from collagen, chitosan, and hyaluronic acid loaded with poly(lactic-co-glycolic acid) microspheres containing kartogenin; while the transition layer was prepared from collagen, chitosan, and silk fibroin loaded with polylysine-heparin sodium nanoparticles containing TGF-β1. Compared with isolated layers, the results of the dual-layer biomimetic hydrogel showed that the defects were completely filled, there was almost no difference between the new cartilage and the surrounding tissue, and the morphology of the cells in the repair tissue was almost in agreement with normal cartilage after 16 weeks. Similarly, Radhakrishnan et al. [78] fabricated a gradient nanoenriched hydrogel using alginate and polyvinyl alcohol with layer-specific nanoparticles (nanohydroxyapatite and nanoparticles based on chondroitin sulfate). This system showed promising results in the healing of osteochondral defects in rabbits, demonstrating integration, neohyaline cartilage, and mineralized neomatrix formation. N. Lin et al. [79] designed double membrane microgels using chemically modified cellulose nanocrystals (cationic CNC) and alginates (anionic). These microgels had an alginate-based outer membrane and an inner membrane formed through electrostatic interactions between cationic and anionic polymers. The dual-membrane structure allowed for the incorporation of two different drugs, each residing in distinct membranes, ensuring codelivery with varied release kinetics. The alginate outer membrane facilitated rapid drug release, while the internal cationic CNC membrane exhibited a sustained drug release profile attributed to the “nano-locking effect.”

Three-dimensional printing is becoming a prominent technology for preparing hydrogels. By making use of their advantages over traditional manufacturing techniques, complex hydrogels can be obtained relatively easily, either by varying the composition of the manufactured hydrogel itself in just one single step or by preparing complex structures, which cannot be achieved by traditional techniques. For example, F. Gao et al. [80] used thermally assisted extrusion-based 3D printing to fabricate a biohybrid gradient hydrogel scaffold for the effective repair of osteochondral defects. The printing ink consisted of a cleavable hydrogen-bonded monomer, *N*-acryloyl 2-glycine and a biopolymer, methacrylated gelatin. The resulting hybrid hydrogel, with appropriate stability and mechanical properties, was suitable for in vivo implantation as a load-bearing scaffold. To improve the repair efficacy, bioactive Mn^2+^ ions were loaded into the top cartilage layer and bioactive glass was incorporated into the bottom subchondral bone layer to construct a gradient hydrogel scaffold. Mn^2+^ ions and bioglass in the printed gradient hydrogel facilitated the chondrogenic and osteogenic differentiation of bone marrow stem cells, respectively. H. Yang et al. [81] reported heterogeneous hydrogels with a tough gel as the skeleton and another soft gel as the matrix, created using a combination of SLA printing and casting. The skeleton hydrogel was polyacrylamide–polyacrylic acid strengthened by the formation of carboxyl-Fe^3+^ coordination complexes, while the stretchable polyacrylamide hydrogel served as the soft matrix. This combined strategy for the development of heterogeneous hydrogels was extended to various hydrogel species, such as poly(*N*-isopropyl acrylamide), poly(hydroxyethyl methacrylate), and gelatin. Multifunctional additives play a vital role in enhancing the capabilities of 3D printed hydrogel constructs. These functional additives include living cells, functional polymers, and particles. Hydrogels that closely resemble the environment of the extracellular matrix can facilitate cell attachment, migration, proliferation, and differentiation. Due to the excellent biocompatibility of hydrogels, bioprinting has been explored using cell-laden bioink to create 3D materials and devices with biological activity [82]. For example, Nadernezhad et al. [83] developed an agarose-based hydrogel loaded with laponite nanosilicates for extrusion-based 3D bioprinting. The cell-laden bioink, incubated above its gelation temperature, was printed in a continuous extrusion-based print at room temperature, showcasing fast gelation and confirming the bioactivity of the printed hydrogels through cell spreading and extension. Kolesky et al. [84] successfully bioprinted thick vascularized tissues from a gelatin–fibrin matrix with high-fidelity micrometer-sized resolution, comprising the parenchyma, stroma, and endothelium. These tissues could be actively perfused with growth factors for extended periods of time, demonstrating significant potential for future human tissue regeneration. Table 1 includes examples previously described as hydrogels with diffusion-controlled release, including materials used for their synthesis and their application.

However, because of their static structure, conventional hydrogels lack responsiveness to environmental changes, limiting their applicability in situations where controlled, on-demand drug release is desired.

### 5.2. Release in Smart Hydrogels

Smart or stimuli-responsive hydrogels represent an innovative advancement in drug delivery technology, as they can respond to specific environmental conditions by altering their physical or chemical properties, thus releasing drugs in a controlled and targeted manner [9,85,86]. These hydrogels are engineered to respond to a variety of external stimuli, including pH, temperature, light, magnetic fields, and ionic concentration, allowing precise control over the drug release process and making them particularly suitable for targeted therapies [87]. Smart hydrogels have expanded the potential applications of hydrogel-based DDS beyond sustained release. They allow for on-demand release triggered by specific physiological or external cues, providing enhanced precision in drug delivery and reducing the likelihood of adverse side effects. Moreover, these hydrogels are increasingly used in combination therapies, where multiple drugs are loaded into a single hydrogel and released selectively in response to different stimuli, allowing for complex treatment regimens tailored to individual patient needs. The versatility of smart hydrogels also extends their applications to fields such as tissue engineering and biosensing, where their adaptive properties provide a responsive environment that can support cellular growth or detect biomolecular signals [9,85,86].

#### 5.2.1. pH-Responsive Hydrogels

pH-sensitive hydrogels are designed to swell or contract in response to changes in pH, making them ideal for targeting areas of the body with distinct pH levels, such as the gastrointestinal tract or tumor microenvironments. In an acidic environment, as commonly found in inflamed tissues or tumors, pH-sensitive hydrogels swell, triggering the release of their drug payload. This type of controlled location-specific release can minimize systemic side effects and improve the efficacy of treatments for diseases such as cancer [28]. Wounds are usually acid environments; therefore, some authors have proposed the use of these types of stimuli-response hydrogels for wound healing [88]. Ninan et al. [89] prepared carboxylated agarose cross-linked with zinc ions and incorporated tannic acid, which allowed for the controlled release of tannic acid in response to acidic pH. This hydrogel demonstrated antibacterial and anti-inflammatory properties, making it highly suitable for wound healing applications by releasing its therapeutic agents in infected, acidic environments. Similarly, Ma et al. [90] described a thermosensitive and pH-responsive hydrogel combining hydroxypropyl chitin cross-linked with ferric ions and tannic acid. In addition to a prolonged antibacterial effect, this hydrogel also demonstrated effective healing capabilities in a mouse wound model, reducing scar formation and supporting tissue regeneration. Other hydrogels were designed in bead form, such as the core–shell structured alginate/carboxymethyl cellulose hydrogel beads prepared by Yan et al. [91]. These beads were designed with an alginate core and carboxymethyl cellulose shell, ensuring mechanical stability at gastric pH while releasing drugs in intestinal pH. They offered a cumulative release rate of 92% at intestinal pH, suitable for anti-inflammatory drugs like indomethacin.

A long-celebrated disparity in pH between healthy tissues (pH ~ 7.4) and tumor tissue (pH ~ 6.5–6.8) offers a mechanistic reflection of the metabolic behavior switched at large in cancer cells; those tend to rely heavily on glycolysis, with overproduction of lactic acid under hypoxic conditions (‘Warburg effect’) [92,93]. Such local acidification may be exploited for pH-responsive DDS design. For example, micelles comprising pH-sensitive functional groups like imine or hydrazone linkages could degrade in an acidic environment and thus release the drug into the tumor microenvironment [94,95]. M. Wu et al. [96] prepared an injectable, self-healing, and pH-responsive composite hydrogel based on Schiff base reactions between aldehyde-functionalized polymers and amine-modified silica nanoparticles, allowing rapid gelation, injectability, and a pH-sensitive gel-sol transition, which is crucial for controlled drug release in cancer therapy and infection treatment. Another example was proposed by J. Qu et al. [97] based on *N*-carboxyethyl chitosan and PEG aldehyde, where the hydrogel presented self-healing and pH-sensitive properties, making it suitable for localized drug delivery in hepatocellular carcinoma. It released doxorubicin in acidic tumor environments, enhancing anticancer efficacy and reducing side effects. The biodegradable, pH-responsive dextran phosphate-based hydrogel loaded with prospidine exhibited selective drug release in acidic tumor environments, showing increased antitumor activity and prolonged therapeutic effects. It was particularly suited for localized therapy of solid tumors [98]. Designed specifically for intratumoral injections, the paclitaxel-loaded peptide hydrogel utilized a pH-sensitive peptide network to release paclitaxel in acidic tumor environments. It improved drug accumulation in tumors, demonstrating prolonged retention and enhanced anticancer efficacy in breast cancer models [99]. In a similar approach, the hydrogel designed with OE polypeptides (polypeptides engineered with specific sequences or chemical modifications that allow them to self-assemble), released gemcitabine and paclitaxel simultaneously in acidic tumor environments, improving the effectiveness of combination therapy by ensuring that both drugs reach the tumor site concurrently [100]. A. Dong et al. [101] developed an injectable pH-sensitive hydrogel with CuMnS nanoenzyme catalyst. This hydrogel was obtained by linking methoxy PEG and cinnamaldehyde with adipic acid dihydrazide. It combined pH-responsive properties with photothermal, photodynamic, and chemodynamic therapies, utilizing a CuMnS nanoenzyme to generate reactive oxygen species under acidic conditions, making it highly effective in multimodal cancer treatments.

Variation in pH along the gastrointestinal path is a key factor in the development of DDS focusing on treatments with active principles whose release is intended to occur once the stomach has passed. One example was offered by Khan et al. [102], who prepared ezetimibe-loaded pH-sensitive PEG/polyacrylic acid hydrogel, which targeted drug release in the small intestine (pH 6–7.4), using a swelling mechanism for sustained drug release. It demonstrated a release efficiency of 80–90% within 24 h, following first-order kinetics, making it ideal for cholesterol absorption inhibitors such as ezetimibe. Polycaprolactone and methacrylic acid graft copolymer hydrogel was designed for radioprotective drug delivery. This hydrogel protected drugs such as amifostine at acidic pH and allowed burst release at intestinal pH (7.4). It improved drug bioavailability and survival outcomes in radiation-exposed models [103]. In the literature, some dual responsive hydrogels can be found. As an example, Bai et al. [104] developed a thermo/pH-sensitive hydrogel made from *N*-succinyl hydroxybutyl chitosan. This hydrogel was dual sensitive to temperature and pH, increasing its adaptability for various drug formulations. It demonstrated significantly higher release rates of model drugs at neutral pH, indicating its potential in targeted drug delivery for oral applications. In another example, Y. Huang et al. [105] prepared a pH/redox-dual responsive hydrogel for magnesium ion delivery. This hydrogel used lysine-based crosslinkers to create a matrix responsive to acidic and reducing environments. It protected the ions at gastric pH (1.2) and efficiently released them at intestinal pH (6.8), showing the potential for the delivery of mineral ions. Table 2 includes the examples previously described as pH-responsive hydrogels, including materials used for their synthesis and their application.

#### 5.2.2. Temperature-Responsive Hydrogels

These hydrogels expand or contract according to temperature variations, which is particularly advantageous for delivering drugs to inflamed or diseased tissues where local temperature can be elevated [127,128,129,130]. For example, temperature-sensitive hydrogel might remain stable at room temperature but swell upon encountering body temperature, thereby initiating drug release at the site of inflammation. Tunning the hydrogels’ composition and degree of crosslinking, it is possible to design ad hoc systems that respond predictably at physiological or pathological temperatures, ensuring efficient release with minimal secondary effects. The mechanism of thermal-triggered drug release is based on the polymer’s lower critical solution temperature (LCST) or upper critical solution temperature (UCST) [130,131]. The hydrogel undergoes a phase transition, altering its structure and permeability and disrupting the encapsulation environment, leading to a controlled burst or sustained release. Injectable thermosensitive hydrogels are an important subclass, as they transition from liquid to gel upon reaching physiological temperatures, allowing for minimally invasive administration and in situ drug release [132,133]. An example was given by M. Gou et al. [106], who prepared an injectable hydrogel from poly(ε-caprolactone)-PEG-poly(ε-caprolactone). It undergoes a sol–gel transition at physiological temperature, forming a nonflowing gel that allowed for the sustained release of hydrophobic drugs like honokiol. Similarly, Sim et al. [107] described the synthesis of a thermosensitive injectable hydrogel based on heparin-bearing poly(ε-caprolactone-co-lactide)-b-PEG-b-poly(ε-caprolactone-co-lactide). This hydrogel, designed for protein delivery, transitions from sol to gel at body temperature. It allowed for sustained release of proteins like lysozyme and exhibited excellent biocompatibility and minimal cytotoxicity in vivo. Liang et al. [108] described a methylcellulose/alginate hydrogel that combined temperature-sensitive methylcellulose with alginate, forming a gel at body temperature for protein drug delivery. It demonstrated effective site-specific release of bovine serum albumin in response to physiological changes in pH and temperature. Another example is the use of thermosensitive poly(ether urethane) hydrogels with pH-sensitive mesoporous silica nanoparticles for dual-responsive localized delivery. It demonstrated fast gelation, tunable release rates, and enhanced drug targeting for localized therapies [109]. Yanev et al. [110] synthesized a thermosensitive biodegradable hydrogel for neurological disorders. Based on PEG, *N*-(2-hydroxypropyl) methacrylamide-mono/dilactate and thiolated hyaluronic acid, this hydrogel was designed for sustained protein release in brain applications, providing controlled delivery for up to three weeks.

It is worth mentioning the development of temperature-sensitive hydrogels for cancer treatment. In this case, hydrogel sol–gel transition at physiological temperatures allows localized drug delivery for cancer treatments such as chemotherapy, photothermal therapy, and immunotherapy. It provides high local drug concentration and sustained release with minimal systemic toxicity [134]. Luo et al. [111] applied chitosan-based hydrogel for localized breast cancer therapy using triptolide-loaded nanostructures. It showed enhanced tumor suppression by combining pro-apoptotic and anti-angiogenesis effects. To combat the same disease, hydrogels obtained from chitosan and agarose loaded with graphene or graphene oxide combined localized drug delivery and photothermal effects to improve the efficacy of breast cancer treatment while minimizing side effects [112]. Also, combining chemo-photothermal therapy, hydrogel obtained from chitosan, and β-glycerin sodium phosphate containing glycyrrhetinic acid-modified graphene oxide was applied. The hydrogel demonstrated temperature-dependent drug release and strong antitumor effects in hepatocellular carcinoma [113]. Al Sabbagh et al. [114] developed a poloxamer-based hydrogel for local chemotherapy in colorectal cancer. This thermosensitive hydrogel integrated poloxamers with 5-fluorouracil. It ensured sustained drug release and improved therapeutic outcomes with minimal systemic exposure. Table 2 includes the examples previously described as temperature-responsive hydrogels, including materials used for their synthesis and their application.

#### 5.2.3. Photo-Responsive Hydrogels

These hydrogels release drugs in response to light exposure, typically ultraviolet (UV) or near-infrared (NIR) light, which provides precise spatial and temporal control over drug release. Photo-responsive hydrogels can be particularly useful in treating conditions such as cancers or localized infections, where targeted light application can trigger localized drug release directly on the lesion site, reducing off-target effects and improving treatment efficacy [10].

In addition to the advantages described of offering precise, effective, and minimally invasive treatments, the use of photo-responsive hydrogels also presents the possibility of multimodal therapies, one of the most prominent options in the current treatment of cancer. In this sense, using polydopamine and alginate as the outer layer and gelatin as the core, fibers obtained by 3D printing allowed for localized and controlled drug release triggered by NIR light, ideal for postsurgical cancer treatment [10]. Z. Zhang et al. [115] developed a photosensitive hydrogel based on silk sericin and chitosan for breast cancer. This hydrogel, encapsulating ROS-sensitive tegafur and protoporphyrin IX, combined photodynamic therapy and chemotherapy. It offered on-demand drug release triggered by NIR light, maximizing therapeutic effects while reducing toxicity. P.-P. He et al. [116] prepared a hydrogel that integrates DNA and Ti3C2Tx-based MXene (atomically thin layers of transition metal carbides, nitrides, or carbonitrides) also for NIR-triggered photothermal-chemo synergistic therapy. Under NIR light, it undergoes a gel-to-solution transition, releasing doxorubicin for efficient localized cancer therapy while reforming the gel matrix upon light removal [117]. Photothermal agarose hydrogel incorporating manganese oxide nanoparticles and chlorin e6 relieved tumor hypoxia and enabled NIR-induced photothermal and photodynamic therapies for enhanced tumor ablation. A Silk fibroin hydrogel was synthetized by S. Gou et al. [118]. This multistimuli-responsive injectable hydrogel released drugs like DOX upon NIR-triggered gel–sol transition. It combined chemo, photothermal, and photodynamic therapies to eliminate tumor masses and extend survival time in tumor-bearing mice. X. Zhu et al. [119] developed a 4-arm-PEG-SH injectable hydrogel, cross-linked with tannic acid and Fe^3+^ complexes, which exhibited intrinsic NIR absorption for synergistic photothermal-chemotherapy and provided enhanced drug uptake and tumor ablation with excellent biocompatibility.

For the treatment of localized infections, an intelligent hydrogel was synthetized integrating pH-sensitive bromothymol blue and NIR-absorbing conjugated polymers, poly{2,5-thiophen-co-[3,6-di(thiophen-2-yl)-2,5-bis(*N,N,N*-trimethylhexan-1-aminiu-m)pyrrolo[3,4-c]pyrrole-1,4(2H,5H)-dione]-bromide-co-4,7-(2,1,3-benzothia-diazole)}, into a thermosensitive matrix based on chitosan. It enabled visual diagnosis of bacterial infections and photothermal eradication of *Staphylococcus aureus* biofilms, making it effective against a broad range of bacteria, including drug-resistant strains [120]. Q. Li et al. [121] prepared a hydrogel using PEG diacrylate as a matrix, polyoxometalate as an acidity-enhanced photothermal agent and 2,2′-azobis[2-(2-imidazolin-2-yl) propane] dihydro-chloride as thermo-responsive initiator. This hydrogel leveraged NIR light to initiate gelation and provide localized photothermal and thermodynamic antibacterial therapy. It was designed for non-antibiotic treatment of subcutaneous infections, targeting resistant bacteria with minimal systemic side effects. Incorporating MnO_2_ nanosheets and CaO_2_ nanoparticles, an alginate-based hydrogel generated oxygen to alleviate biofilm hypoxia and enhance photodynamic antibacterial activity. It effectively eradicated bacterial biofilms and promoted healing in infection models [122]. Manganese oxide nanoparticles were also applied for the preparation of injectable hydrogel, combining redox and light responsiveness for treating multidrug-resistant bacteria and healing infected wounds. It delivered photothermal hyperthermia and sustained drug release, significantly accelerating wound healing while battling bacterial infections [123]. Also incorporating nanoparticles, Deng et al. [124] described agarose-based photothermal hydrogel containing tannic acid-Fe^3+^ nanoparticles, creating an eco-friendly hydrogel with near-total bacterial eradication with exposure to NIR light. It demonstrated excellent in vitro and in vivo antibacterial activity, aiding in the treatment and healing of wound infections. Combining copper nanoparticles for photothermal conversion and sustained Cu^2+^ ion release to combat both Gram-positive and Gram-negative bacteria, the gelatin-based hydrogel proposed by B. Tao et al. [125] accelerated healing in infected wounds while reducing inflammation and promoting angiogenesis. Using CuS nanoparticles, the hydrogel obtained from hyaluronic acid and Fe^3+^-EDTA complexes combined chemodynamic therapy and low-temperature photothermal therapy for infected wounds; it sterilized effectively while minimizing tissue damage, supporting robust healing [126]. Table 2 includes the examples previously described as photo-responsive hydrogels, including materials used for their synthesis and their application.

#### 5.2.4. Magnetically Responsive Hydrogels

By embedding magnetic nanoparticles into a hydrogel matrix, they can respond to external magnetic fields, allowing for noninvasive, remote controlled drug release. This approach has shown promise in delivering drugs to deep tissues or specific organs, where the magnetic field can guide the hydrogel to the target site and initiate drug release through localized heating or structural changes induced by the field [21]. For these reasons, this is one of the most investigated approaches for the treatment of cancer. In this sense, different hydrogels have been investigated for the sustained release of doxorubicin. For example, dextran microgels with embedded Fe_3_O_4_ nanoparticles exhibited dual responsiveness to pH and magnetic fields. These hydrogels enabled the controlled release of doxorubicin [135]. Similarly, a biocompatible silk fibroin hydrogel integrating Fe_3_O_4_ nanoparticles was designed for sustained doxorubicin release under magnetic fields, reducing systemic toxicity [136]. A hydrogel based on alginate, gelatin, and Fe_3_O_4_ nanoparticles demonstrated pH-dependent release of doxorubicin, making them ideal for localized chemotherapy [137]. Cao et al. [138] prepared a hydrogel combining poly(*N*-isopropylacrylamide) and alginate with graphene oxide and Fe_3_O_4_ nanoparticles. It releases doxorubicin upon exposure to magnetic fields, light, and pH changes, offering controlled drug delivery for cancer therapy. Dai et al. [139] described a catechol–metal coordinated nanocomposite hydrogel. This hydrogel used dopamine-conjugated hyaluronac crosslinked with Fe_3_O_4_ nanoparticles. It allowed on-demand, magnetic field-triggered release of doxorubicin for combination chemo-hyperthermia cancer therapy. Or a hydrogel based on tragacanth gum and acrylic acid-based hydrogel used Fe_3_O_4_ nanoparticles to release doxorubicin in response to environmental triggers [140]. However, other active principles have also been delivered. For example, the tragacanth gum, *N*-isopropylacrylamide, and 3-(trimethoxysilyl) propylmethacrylate hydrogel incorporating Fe_3_O_4_ nanoparticles delivered anticancer drugs like methotrexate with enhanced magnetic responsiveness [141]. Carboxymethyl cellulose/β-cyclodextrin/chitosan hydrogels loaded with Fe_3_O_4_ magnetic nanoparticles demonstrated pH-sensitive methotrexate release, with enhanced release under acidic conditions. This makes them suitable for targeted delivery in tumor environments [142].

The use of magnetic field-sensitive hydrogels has also been widely investigated for wound healing because of their capacity to selectively locate and deliver drugs. P. Wang et al. [143] developed a hydrogel combining cobalt ferrite nanoparticles with polyvinyl alcohol, using tannin as a bridging agent. It responded to static magnetic field, enhancing cell adhesion, proliferation, antibacterial activity, and angiogenesis, thereby accelerating wound healing. Integrating MXene-wrapped magnetic Fe_3_O_4_@SiO_2_ nanoparticles, this poly(*N*-isopropyl acrylamide)-alginate dual-network hydrogel delivered drugs in a photo- and magnetoresponsive manner. It reduced drug toxicity and promoted the healing of deep chronic wounds, as demonstrated in full-thickness and infected wound models [144]. Using Fe_3_O_4_-based magnetic microspheres from natural polysaccharides, the chitosan-cellulose hydrogel prepared by Z. Wang et al. [145] offered self-healing, thermal reversibility, and controlled drug release. It was effective for delivering drugs in wound healing applications while maintaining mechanical stability. The starch-based hydrogel proposed by Nezami et al. [146] incorporated Fe_3_O_4_ nanoparticles and itaconic acid grafted onto starch, enabling pH-sensitive and magnetically enhanced drug release. It was highly effective in wound healing and demonstrated a controlled release of guaifenesin.

But the use of magnetically responsive hydrogels is not reduced to cancer treatment or wound healing. Examples of magnetic-responsive hydrogels are also found for the treatment of neurological disorders [147]. In this sense, injectable alginate hydrogel with magnetic short nanofibers for neural regeneration was described by Ghaderinejad et al. [148]. This hydrogel was composed of alginate incorporating magnetic polycaprolactone short nanofibers containing superparamagnetic iron oxide nanoparticles. It demonstrated the ability to align fibers under an external magnetic field, promoting neural differentiation and enhancing the bioactivity of encapsulated olfactory ecto-mesenchymal stem cells. This hydrogel showed promise as a minimally invasive scaffold for neural tissue repair. The system designed by Kang et al. [149] utilized a thermosensitive injectable hydrogel based on poly(lactic acid-ethylene glycol-lactic acid) incorporating drug-loaded micelles and water-dispersible ferrimagnetic iron oxide nanocubes. After injection into a resected glioblastoma site, the hydrogel gelated at body temperature, serving as a deep intracortical drug reservoir. Magnetic fields induced hyperthermia and enhanced drug diffusion, effectively targeting residual glioblastoma cells. This approach demonstrated significantly suppressed tumor growth and improved survival in a mouse model of glioblastoma.

However, the investigation of magnetic-responsive hydrogels is currently so widely spread in the scientific community that other different applications can be found in the literature. For example, Bao et al. [150] described a chitosan/β-glycerophosphate hydrogel loaded with superparamagnetic iron oxide nanoparticles to provide magnetic stimulation for the repair of the vagus nerve. Under a mild magnetic field (~100 mT), it reduced heart rate and improved vagus nerve activity. Four weeks of magnetic stimulation (20 Hz, 5 min, three times daily) post-myocardial infarction significantly enhanced cardiac function, reduced infarct size, and suppressed inflammation in myocardial infarction models in rats, demonstrating promising noninvasive treatment for myocardial infarction. Mahdavinia et al. [151] designed a carboxymethyl cellulose/montmorillonite hydrogel for colon targeting. This magnetic nanocomposite hydrogel, incorporating carboxymethyl cellulose, acrylamide, *N,N*′-methylene bis acrylamide (as crosslinking agent) and magnetic (Fe_3_O_4_) montmorillonite, was designed for colon-specific drug delivery. It released diclofenac effectively in response to pH and magnetic field stimuli, targeting localized drug delivery in the gastrointestinal tract. Liposomes loaded with ferulic acid were included in magnetic gelatin hydrogels also containing Fe_3_O_4_ magnetic nanoparticles. It enabled precise, magnetically modulated drug release, suitable for cell development and faster tissue regeneration [152].

In summary, the development of smart hydrogels has transformed the landscape of drug delivery by enabling controlled, targeted, and personalized treatment options. As research advances, new types of stimuli-responsive hydrogels continue to emerge, offering exciting possibilities for future therapeutic applications. Table 3 includes the examples previously described as magnetically responsive hydrogels, including materials used for their synthesis and their application.

#### 5.2.5. Redox-Responsive Systems

Redox-responsive have been studied to utilize the intracellular redox environment, such as NADP^+^/NADPH, O_2_/$O_{2}^{-}$ and glutathione (GSH)-reduced glutathione (GSSG), particularly the enhanced levels of GSH in cancer cells, 4–5 times higher than normal ones [159]. This pH imbalance, added to the presence of disulfide linkage within the polymer structure, gives the prospect of selective drug release in such redox-active locales. An example of a redox-sensitive hydrogel using the thiol-disulfide exchange method was reported by Kilic Boz et al. [153], who developed linear telechelic PEG-based polymers combined with pyridyl disulfide units and thiol-terminated tetraarm PEG polymers. This resulted in the formation of macroporous hydrogels with high water absorption (>85%), and self-healing because of disulfide linkages. These hydrogels disintegrated in thiol-containing environments such as GSH, enabling the controlled release of bovine serum albumin. Cytocompatibility tests verified their suitability for biological drug delivery purposes, allowing drug release as needed. D. Huang et al. [154] produced microgels that were sensitive to redox environments by combining bis(2-methacryloyloxyethyl) disulfide, PEG, and 2-(diisopropylamino) ethyl methacrylate. The microgels inhibited tumor cell proliferation in vitro and reduced tumor growth in living organisms, showing potential for treating cancer. Based on the NADP^+^/NADPH, W. Tao et al. [155] created a hydrogel containing calcium selenite/L-arginine nanospheres, glucose oxidase, and 6-aminonicotinamide in sodium alginate. The selenite and 6-aminonicotinamide synergistically triggered intracellular NADPH exhaustion by promoting cystine metabolism-mediated NADPH consumption and inhibiting NADPH generation, respectively. This injectable hydrogel formed under natural conditions stimulated immunogenic cell death in tumor cells by increasing reactive oxygen species (ROS) and reactive nitrogen species (RNS). Robby et al. [156] developed a O_2_/$O_{2}^{-}$ responsive system. The incorporation of ROS-sensitive MnO_2_ initially suppressed the calcium phosphate mineralization of polyacrylic acid via carboxyl-Ca^2^⁺ complexation. In the presence of elevated ROS levels typically found in cancer cells, the MnO_2_ was cleaved into Mn^2^⁺ ions, triggering the mineralization process and leading to the formation of a PAA-MnO_2_ mineralized hydrogel. In vivo studies conducted on tumor-bearing mice demonstrated that the PAA-MnO_2_ hydrogel effectively formed at tumor sites, mainly due to its robust ROS-scavenging capabilities. Similarly, a novel hydrogel was prepared by S. Wang. et al. [157] involving MnO_2_ nanosheets coated with ε-polylysine and insulin-loaded micelles formed by self-assembled aldehyde Pluronic F127. Taking advantage of the combined effects of ε-polylysine and the “nanoknife-like” structure of MnO_2_ nanosheets, the resulting hydrogel demonstrated remarkable antimicrobial efficacy against multidrug-resistant bacteria. Furthermore, the MnO_2_ nanoenzyme effectively modulated the oxidative wound microenvironment by catalyzing the conversion of endogenous H_2_O_2_ into O_2_. Simultaneously, the pH- and redox-responsive hydrogel facilitated the controlled, sustained, and localized release of insulin, contributing to blood glucose regulation. Polo et al. [158] prepared a hydrogel integrating graphene derivatives and cerium oxide nanoparticles, forming a three-dimensional scaffold that enhanced neural stem cell adhesion, migration, and differentiation. It supported the differentiation of neural stem cells into neuronal, astroglial, and oligodendroglial cells, providing a promising platform for neural tissue engineering and regenerative therapies. Table 3 includes the examples previously described as redox-responsive hydrogels, including materials used for their synthesis and their application.

#### 5.2.6. Enzyme-Triggered Systems

Enzyme-sensitive hydrogels represent a versatile and innovative tool for controlled drug delivery as a result of their ability to respond specifically to enzymatic activity in targeted environments. These hydrogels leverage the elevated expression or activity of specific enzymes, such as matrix metalloproteinases (MMPs), glucose oxidase (GOx), alkaline phosphatase (ALP), hyaluronidase or β-galactosidase, often associated with pathological conditions like cancer or inflammation. This precise responsiveness allows the release of therapeutic agents only when and where needed, minimizing systemic side effects and enhancing therapeutic efficacy [160]. Moreover, enzyme-sensitive hydrogels can incorporate labile linkages or crosslinks that selectively degrade in the presence of overexpressed enzymes in diseased tissues, such as tumor microenvironments, enabling localized and on-demand drug release [161]. Their biodegradability, biocompatibility, and ability to respond under mild physiological conditions further make them ideal candidates for DDSs in sensitive therapeutic scenarios [162]. These advantages highlight the potential of enzyme-sensitive hydrogels to revolutionize targeted therapies, especially in cancer treatment and regenerative medicine.

As mentioned above, hydrogels that respond to different specific enzymes have been developed. Hydrogels sensitive to glucose have demonstrated significant utility in the preparation of specific self-regulating liberation systems. GOx is able to convert glucose into gluconic acid [160]. This pH decrease triggers the release of insulin from pH-responsive hydrogels. As an example, hydrogels synthesized from *N,N*-diethylaminoethyl methacrylate and 2-hydroxypropyl methacrylate, crosslinked with a polyacrylamide membrane immobilizing GOx, have shown promising results [163]. Low pH membrane induced protonation of amino groups causing drug release, leading to hydrogel expansion and increasing permeability of membrane to insulin. Similarly, concanavalin A is a natural carbohydrate-binding protein and forming a tetrameric structure that can bind to four glucose molecules, acting as a macromolecular crosslinker. D. Dong et al. [164] developed Cu_2_O/Pt hydrogels through the mixture of Cu_2_O/Pt nanocubes with alginate and hyaluronic acid. The hydrogel utilized GOx to convert glucose, producing gluconic acid while decreasing the pH in the wound site. In acidic conditions, Cu_2_O/Pt nanocubes react with H_2_O_2_ to release hydroxyl radicals, which leads to antibacterial effects through ROS-based mechanisms. Cu ions released from the hydrogel also enhance the expression of vascular endothelial growth factor, leading to the stimulation of endothelial cell proliferation and angiogenesis, both essential for wound healing. In vivo studies conducted on diabetic rats infected with *S. aureus* showed that the Cu_2_O/Pt hydrogel sped up wound healing, raised epithelial thickness, and improved the regeneration of hair follicles and sebaceous glands. Xian et al. [165] developed a hydrogel for glucose monitoring and insulin delivery. It consisted of a glucose oxidase-responsive hydrogel based on phenylboronic acids and diols that integrated dynamic covalent crosslinking to enable insulin release in response to glucose levels. It offered a stable and injectable platform to manage blood glucose with minimal daily interventions.

Concanavalin A can be integrated into the hydrogel matrix and can release insulin depending on glucose levels [166]. K. Lin et al. [167] created a hydrogel that responds to glucose, using pullulan and modifying concanavalin A covalently derivative with COOH groups, enabling smart and regulated insulin release with changes in levels of glucose. The hydrogel expands and discharges insulin upon stimulation. Elevated glucose levels lead to increased insulin release; however, insulin release decreases when glucose levels are low. But some other enzyme-sensitive hydrogels have been developed to deliver active principles different from insulin and using a different strategy.

MMPs are a group of endopeptidases capable of breaking peptide bonds. They are involved in adhesion, survival, proliferation and differentiation, migration, and intercellular interactions, among other functions. Tumor cells secrete MMPs and other proteolytic enzymes into the extracellular matrix to degrade its components and facilitate tumor growth. High MMP activity, particularly in tumor tissues, makes these enzymes ideal as biological triggers for enzyme-responsive anticancer DDSs [160]. Based on the response to these enzymes, C. Cai et al. [168] developed a hydrogel consisting of carboxymethyl chitosan with MMP-2 as crosslinker and polycaprolactone. This hydrogel was designed to form electrospun fibers to release siRNA in response to MMP-2 activity. This system presented a unidirectional design to avoid interference in tissues such as tendons, effectively inhibiting fibroblast proliferation, reducing tendon adhesions, and silencing fibrosis gene transforming growth factor-β1, thus reducing side effects in sensitive tissues. W. Chen et al. [169] synthesized an MMP-2/9-responsive hydrogel system based on tetra-PEG that delivered therapeutic factors (carbon dots coupled with interleukin-4 plasmid DNA) to regulate the immune microenvironment after myocardial infarction. The hydrogel responded to MMP activity in injured tissue, triggering the release of encapsulated agents, which reduced inflammation and improved cardiac repair. Systems sensitive to MMP-13 were described by T. Zhou et al. [170], who prepared a hydrogel microsphere system to treat osteoarthritis by combining ROS scavenging, MMP-13 sensitivity, and hypoxia-responsive drug release. The system used sulfonated azocalix[4]arene as a host molecule to load the anti-inflammatory drug hydroxychloroquine, forming a host–guest complex that synergistically reduces inflammation. Sulfonated azocalix[4]arene was modified with a methacryloyl group to create a covalently crosslinked hydrogel network with methacrylated hyaluronic acid. An MMP-13-sensitive peptide with thiol groups was incorporated into the hydrogel, enabling on-demand drug release and degradation in response to overexpressed MMP-13 in the osteoarthritis microenvironment, to alleviate disease progression.

ALP is an enzyme found throughout the body that catalyzes the hydrolysis of phosphate esters at an alkaline pH (optimal around pH 8–10). This enzymatic activity results in the release of inorganic phosphate and alcohol from the substrate through a coordinated process facilitated by metal ions and nucleophilic catalysis. This enzymatic activity results in the release of inorganic phosphate and alcohol from the substrate through a coordinated process facilitated by metal ions and nucleophilic catalysis [171,172]. ALP is crucial in several physiological processes, including bone mineralization, lipid transport, and cellular signaling. By breaking down pyrophosphate, a natural bone mineralization inhibitor, ALP facilitates hydroxyapatite deposition in bones, which forms the basis for its use in treating hypophosphatasia [171,172]. Additionally, ALP’s ability to detoxify lipopolysaccharides by dephosphorylation highlights its anti-inflammatory properties, making it a promising therapeutic for sepsis, inflammatory bowel disease, and acute kidney injury by reducing systemic inflammation. Its phosphate-releasing activity further contributes to tissue regeneration and wound healing, where ALP promotes mineral formation for bone repair. Therefore, ALP’s responsiveness to phosphate substrates is also exploited in the development of ALP-sensitive hydrogels [173,174]. In this sense, N. Li et al. [175] developed a chitosan membrane incorporating a responsive hydrogel to ALP for the treatment of periodontitis. This hydrogel was prepared from phosphorylated polyester (polyphosphoester-b-PEG-b-polyphosphoester) and minocycline hydrochloride, releasing antibiotic and osteogenic drugs in response to ALP activity. Studies demonstrated that drug release in vitro/in vivo from this membrane enhanced osteogenesis and periodontal treatment when it comes to fighting infection. Y. Gao et al. [176] created a prodrug by combining paclitaxel with the phosphorylated peptide, taking advantage of ALP found in high levels in cancer cells to activate the drug in a specific location. ALP removed the phosphate group from the inactive drug, turning it into a substance that formed a hydrogel at the affected area. This allowed the controlled and continuous release of medication. This approach significantly boosted paclitaxel’s ability to dissolve in a significant way, all while keeping its effectiveness in fighting tumors, bringing together the transportation of the drug and its therapeutic effects within one single molecule. The method offered a worldwide strategy for administering hydrophobic medications with increased accuracy and decreased overall side effects.

Hyaluronidases are a group of enzymes that catalyze the breakdown of hyaluronic acid (HA), a major component of the extracellular matrix (ECM), into smaller fragments. Hyaluronidases cleave HA through hydrolysis (hyaluronan degradation), resulting in decreased viscosity and improved permeability within tissues. Hyaluronidase-sensitive hydrogels have special relevance in cancer therapy. They degrade selectively in the tumor microenvironment where hyaluronidase expression is upregulated, allowing localized delivery of chemotherapeutic agents. For example, Fiorica et al. [177] described a HA/cyclodextrin-based injectable hydrogel for local doxorubicin delivery to solid tumors. In vitro studies demonstrated that the unique physicochemical characteristics of the hydrogel ensure a prolonged release of doxorubicin, effectively inhibiting the growth of colorectal carcinoma micromasses cultured under 3D conditions. In vivo experiments further validated the hydrogel’s efficacy, showing a significant reduction in tumor mass within the animal model while avoiding cytotoxic effects in nontarget organs. Vildanova et al. [178] prepared biodegradable hydrogels based on chitosan and pectin for the delivery of cisplatin. The prolonged release of cisplatin was shown to be dependent on the polymers’ concentrations. In any case, the degradability of the hydrogels in the presence of hyaluronidase enhanced drug delivery specifically in tumor microenvironments, where hyaluronidase activity is elevated. But other applications have been explored. For wound healing, Hong et al. [179] synthesized a hemostatic adhesive hydrogel that effectively controlled bleeding and minimizes abnormal tissue adhesion following hemostasis. A photo-crosslinked hydrogel was developed using methacrylate-modified gelatin and *N*-(2-aminoethyl)-4-(4-(hydroxymethyl)-2-methoxy-5-nitrosophenoxy) butanamide conjugated to hyaluronic acid. This hydrogel exhibited outstanding mechanical strength and tissue adhesion properties, enabling rapid hemostasis. Upon UV photo-activation, the hydrogel polymerized and adhered within seconds, creating a robust bond to wet biological tissue surfaces. The action of hyaluronidases could also be explored for tissue engineering. For example, S. Zhou et al. [180] prepared an injectable, self-healing, and multiple responsive histamine-modified hyaluronic acid hydrogel. The presence of the imidazole moiety allowed a dynamic coordinative bonding with metal ions. Using Zr^4+^, the obtained hydrogel presented responsiveness when exposed to a weak alkaline environment and hyaluronidase, inhibiting bacterial growth and biofilm formation. This behavior highlights this hydrogel as an implantable material for tissue engineering. Table 4 includes the examples previously described as enzyme-responsive hydrogels, including materials used for their synthesis, the enzymes to which they are sensitive and their application.

## 6. Nanogels as Advanced DDSs

**Figure 6:**
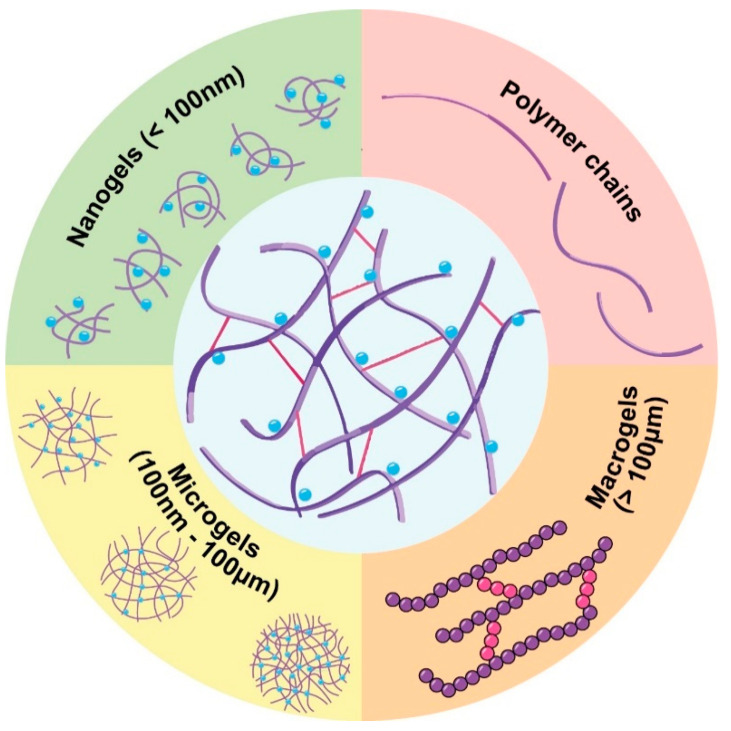
Classification of gels according to their size.

Nanogels are nanoscale hydrogels meticulously engineered for targeted and controlled drug delivery. These nanoscale constructs combine the high water content and biocompatibility of traditional hydrogels with the unique advantages of nanoparticles, such as enhanced penetration, prolonged retention in tissues, and responsiveness to stimuli [181,182,183]. Typically ranging from 20 to 100 nm (Figure 6), their small size allows them to penetrate tissues and accumulate at target sites. This is particularly advantageous in applications demanding high specificity, such as oncology and neurological treatments [181,184,185]. The increasing interest in nanogels stems from their adaptability for incorporating hydrophilic and hydrophobic drugs and responding to environmental or biological signals such as pH, temperature, or enzymatic activity [186]. This flexibility has led to diverse applications, ranging from cancer therapy and neurological treatments to immunotherapy and vaccine delivery. Recent advances in fabrication techniques, such as chemical crosslinking and self-assembly, further improve their potential for personalized medicine.

### 6.1. Physical and Chemical Properties of Nanogels

Nanogels exhibit a high surface area-to-volume ratio, which enhances their capacity for drug loading and facilitates interactions with target tissues. Their small size allows them to bypass biological barriers more effectively than larger particles, such as traditional hydrogels, and enables prolonged circulation in the bloodstream. This feature is especially critical in cancer therapy, where nanogels can accumulate passively in tumor tissues through the enhanced permeability and retention (EPR) effect, a phenomenon by which leaky vasculature in tumor tissue allows nanoparticles to penetrate and remain at the site [8,187]. The structure of nanogels can be designed in various formats, including spherical, core–shell, and multilayered configurations. For instance, core–shell nanogels consist of an internal core that encapsulates the therapeutic agent and an outer shell that can be engineered to respond to specific stimuli, such as pH or temperature, to control the release profile. This structural flexibility enables customization of the nanogel properties to meet specific therapeutic requirements, such as stability, degradation rate, and response to environmental stimuli [9,188]. The unique design of nanogels also supports surface modifications with targeting ligands, such as antibodies or peptides, which further enhance their specificity for diseased tissues or cellular targets. Additionally, nanogels are designed to exhibit high swelling capacity, enhancing drug loading and release efficiency. This property, combined with the possibility of fine-tuning their chemical composition, enables nanogels to maintain stability under various physiological conditions and release drugs in a controlled manner, a feature crucial for applications where gradual release is essential [189].

Nanogels possess several unique characteristics that distinguish them from other advanced drug delivery systems like liposomes and micelles. While liposomes and micelles offer excellent biocompatibility, the encapsulation of both hydrophilic and hydrophobic drugs, and enhanced circulation times via surface modifications, their structural stability can be compromised under physiological conditions, leading to premature drug leakage [190]. In contrast, hydrogels and nanogels, due to their three-dimensional polymeric networks, provide superior stability and enable sustained or stimuli-responsive drug release, ensuring higher control over drug delivery kinetics [191]. Additionally, the high water content of hydrogels allows them to mimic the extracellular matrix, which is particularly advantageous for applications in tissue engineering and wound healing. However, while the synthesis of liposomes and micelles is well-established and scalable, hydrogels and nanogels face challenges with batch-to-batch variability and cost-effectiveness during large-scale production. By combining the structural advantages of hydrogels/nanogels with the versatility of liposome or micelle technology, future research may explore hybrid systems to overcome the limitations of individual platforms.

### 6.2. Preparation Methods of Nanogels

Nanogels are synthesized using the same general methods previously described for hydrogels. For example, Dispenza et al. [192] highlighted the utility of radiation-induced crosslinking to produce sterile multifunctional nanogels based on chemical crosslinking. Atallah et al. [193] encapsulated the antimetabolite pemetrexed and honokiol, a natural polyphenol, into self-assembled nanogels obtained from hydrophilic proteins, lactoferrin, and carboxy methyl cellulose. However, this method generates lower mechanical stability compared to that of chemically crosslinked nanogels. Regarding polymerization techniques, X. Wang et al. [194] developed poly(hydroxyethyl methacrylate) nanogels using an emulsion polymerization method for effective oral insulin delivery. The nanogels were synthesized to encapsulate insulin and promote its absorption through the gastrointestinal tract. Photopolymerization involves initiating polymerization using light. It allows for precise spatial and temporal control over nanogel formation. Typically, UV or visible light is used to activate a photoinitiator, leading to crosslinking of the polymer matrix. It allows high control over the polymerization process, minimal side reactions, and environmental responsiveness; however, it requires specialized equipment and may limit scalability. Z. Yang and J. Ding [195] demonstrated the use of photopolymerization to create thermosensitive nanogels with unique sol–gel transition properties.

However, it is worth mentioning the use of microfluidic systems, a cutting-edge approach for the preparation of nanogels. Microfluidic systems offer unparalleled control over particle size, composition, and structural properties [196]. They enable fine-tuning of flow rates, mixing conditions, and reaction parameters to achieve nanogels with consistent size and morphology. This level of control is crucial for applications requiring monodisperse nanogels, such as targeted drug delivery. A wide range of materials, including natural (e.g., chitosan, alginate) and synthetic (e.g., PEG) polymers, can be used to produce nanogels with tailored properties. In addition, the automated nature of microfluidic systems ensures high reproducibility in nanogel synthesis, reducing batch-to-batch variability. Continuous-flow microfluidics facilitates large-scale production of nanogels without compromising uniformity. Therefore, these systems take advantage of the manipulation of fluids at the microscale to fabricate nanogels with uniform characteristics, making them highly suitable for applications in drug delivery, tissue engineering, and diagnostics [40,196].

**Figure 7:**
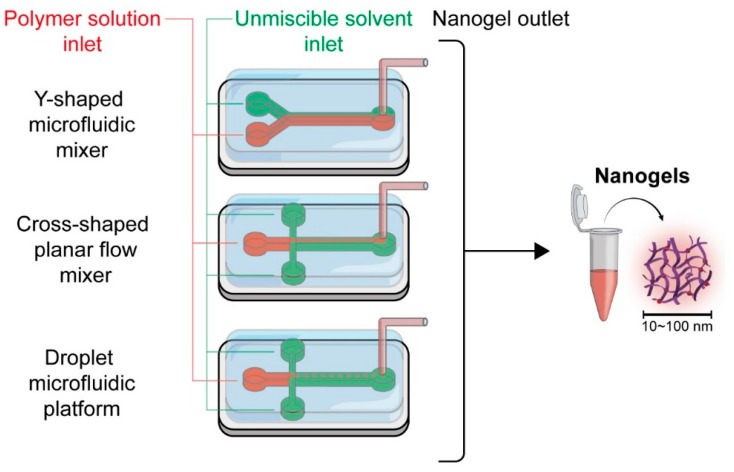
Microfluidic systems used for the preparation of nanogels.

Different techniques are available for the preparation of nanogels. The most widely investigated are droplet-based microfluidics and hydrodynamic focusing. Droplet-based microfluidics (Figure 7) are based on the formation of droplets of a precursor solution in a continuous phase (e.g., oil or water). These droplets act as microreactors, where crosslinking or polymerization occurs to form nanogels. This technique provides precise control over the size and shape of nanogels, especially for synthesizing nanogels with encapsulated drugs or biomolecules. In the hydrodynamic focusing (Figure 7), precursor solutions are focused into narrow streams by co-flowing immiscible fluids, enabling controlled polymerization or crosslinking to form nanogels. It allows nanogels with ultra-uniform size distributions [40,196].

### 6.3. Drug Loading and Release Mechanisms of Nanogels

Hydrogels and nanogels share similarities in their drug-loading mechanisms, including physical encapsulation, electrostatic interactions, and covalent attachment, making them suitable for delivering a wide range of therapeutic agents such as small molecules, proteins, and nucleic acids [197]. Both systems can be engineered for stimuli-responsive release triggered by factors like pH, temperature, or enzyme activity, and are often composed of biocompatible and biodegradable materials [41]. However, they differ significantly in scale and application. Hydrogels, being macroscopic in size, offer larger drug-loading capacities and are ideal for sustained and localized drug release, commonly used in wound healing, implants, and tissue engineering [197]. In contrast, nanogels, with their nanoscale size and high surface area-to-volume ratio, provide faster diffusion and enhanced targeting capabilities, often functionalized with ligands or antibodies for systemic drug delivery [198]. Nanogels are particularly advantageous for applications requiring precision, such as crossing biological barriers (e.g., the blood–brain barrier) or targeting tumors, while hydrogels excel in bulk therapeutic delivery and prolonged drug release [199]. These distinctions allow both hydrogels and nanogels to fulfill complementary roles in DDSs, depending on the therapeutic need. Table 5 offers a summary of the examples used to show the different release mechanisms for nanogels.

#### 6.3.1. Diffusion-Controlled Release

In this approach, drug molecules diffuse slowly from the nanogel matrix as it swells or degrades, providing steady release over time. This mechanism is particularly useful in chronic disease management, where sustained drug levels are necessary for therapeutic efficacy. Y. Zhang et al. [200] prepared dendritic nanogels based on polyethylene glycol and 2,2-bis(hydroxymethyl)propionic acid displaying allyl functional groups UV-crosslinked by monomeric thiols. This simple approach allowed the obtainment of allyl reactive nanogel precursors in a multigram scale that can be further modified to modulate their internal properties for better specific interaction with each therapeutic cargo among the selected chemotherapeutic agents (doxorubicin, gemcitabine and methotrexate).

##### 6.3.2. pH-Responsive Release

Similarly to smart hydrogels, nanogels can be engineered to respond to specific physiological or external stimuli, such as pH, temperature, or magnetic fields. For example, pH-sensitive nanogels are designed to release drugs selectively in acidic environments, such as the tumor microenvironment, ensuring targeted delivery and minimizing the impact on healthy tissues [224]. J.-Z. Du et al. [201] prepared a negatively charged nanogel based on poly(2-aminoethyl methacrylate hydrochloride) modified with 2,3-dimethylmaleic anhydride to produce amide and carboxylic groups, thus increasing the loading efficiency of positively charged doxorubicin hydrochloride at physiological pH. When the loaded nanogel interacted with the acidic environment surrounding the tumor, the cargo was released as the nanogel obtained positively charged. Ju et al. [202] developed a pH dependent swelling–shrinking nanogel containing a crosslinked polyelectrolyte core, composed by *N*-lysinal-*N*′-succinyl chitosan and poly(*N*-isopropylacrylamide), and a crosslinked bovine serum albumin shell. This preparation was capable of quickly dosing the encapsulated doxorubicin in the HepG2 tumor cells showing efficient cytotoxicity. Duan et al. [203] combined pH-responsive nanogel strategy to specifically deliver oridonin to hepatic HepG2 by decorating the nanogel carrier with galactose moieties attached to the chitosan-*graft*-poly(*N*-isopropylacrylamide) polymer precursor. Another interesting improvement related to this release approach was investigated by Q. Song et al. [204], using a biomimetic erythrocyte coating allowing a better infiltration and circulation time before decomposing. The nanogel was prepared with hydroxypropyl-β-cyclodextrin acrylate and charged chitosan derivatives to synergically deliver the antineoplasic agent paclitaxel and the immunotherapeutic agent interleukin-2.

Finally, a combination of this approach and some other physicochemical-dependent phenomena was used for a better performance. Y. Qu et al. [205] fabricated a dual pH/redox-responsive nanogel based on methacrylic acid and *N*,*N*′-methylenebisacrylamide, containing covalently linked camptothecin by a disulfide linker. This system was capable of releasing the drug “on-demand” accelerated by the increased concentration of GSH and the lower pH value present in the tumor cell and tumor tissue environment. In the same spirit, an injectable dual pH/thermo-responsive self-assembled micellar nanogel based on mPEG2000-isopropylideneglycerol and chitosan was synthesized by D. Chen et al. [206], owning a sol–gel phase transition stimulated by body temperature and that could be degraded under low pH conditions to liberate the encapsulated paclitaxel on the tumor tissue.

#### 6.3.3. Thermal-Responsive Release

The unique thermoresponsive properties of nanogels allow them to swell or shrink in response to temperature modifications by exploiting localized hyperthermia or natural temperature gradients within the body [225,226]. Most of the nanogels prepared as thermal-responsive materials are based on poly(*N*-isopropyl acrylamide) (PNIPAM). This polymer presents temperature-responsive behavior around its LCST, approximately 32 °C. At temperatures below the LCST, the polymer remains hydrophilic, maintaining its solubility in water. However, when the temperature exceeds the LCST, the polymer undergoes aggregation and displays hydrophobic properties, leading to a shrinking effect on the system. Giulbudagian et al. [207] described a PNIPAM-based hydrogel to treat inflammatory skin conditions such as psoriasis. To address the deficiency in the stratum corneum barrier, this nanogel containing etanercept was developed. Triggered by changes in temperature, the formulation effectively delivered the drug into the stratum corneum and the viable epidermis, resulting in improved anti-inflammatory activity. Hajebi et al. [208] synthesized hybrid core–shell nanogels composed of NIPAM and vinyl-modified silica loaded with doxorubicin. Drug release was observed to be more pronounced at temperatures below the LCST point. They demonstrated biocompatibility and were able to deliver the drug in a controlled manner to tumor tissues, as evidenced by cytotoxicity studies of the HeLa cell line. Thermoresponsive nanogels have also been employed for gene delivery. Genetic skin diseases that result in reduced protein synthesis require localized protein substitution. Due to the physicochemical limitations of proteins for topical delivery, temperature-responsive nanogels prepared from PNIPAM-polyglycerol were developed by Witting et al. [209] to deliver transglutaminase 1. These nanogels had a thermal trigger point of 35 °C, making them suitable for cutaneous delivery based on the skin’s natural temperature gradient. At temperatures ≥ 35 °C, the nanogels are reduced in size, leading to increased protein release without altering the protein’s structure or activity. The formulation effectively penetrated the skin, particularly in barrier-deficient models, efficiently delivering transglutaminase 1 and restoring barrier function in models deficient in proteins.

#### 6.3.4. Photo-Responsive Release

Nanogels with photosensitive elements offer an innovative approach to controlled drug delivery, using light as a noninvasive trigger. These systems incorporate materials such as azobenzene derivatives, spiropyran, or photo-cleavable linkers, which undergo conformational or chemical changes upon light exposure. For instance, UV-responsive nanogels have been developed using azobenzene crosslinkers, enabling the release of doxorubicin upon UV irradiation. S. Chen et al. [210] described a study focusing on multistimuli-responsive nanogels based on polyacrylic acid derivatized with spiropyran components, enabling UV-triggered swelling and controlled release of doxorubicin, enhancing therapeutic effects. Similarly, nanogels incorporating gold nanoparticles respond to NIR light, which penetrates deeper into tissues, making them ideal for cancer therapy. Y. Zhang [211] et al. prepared a polyacrylic acid and DNA-azobenzene-based nanogel system that acted as a light-fueled nanopump for controllable drug release. The system used upconversion nanoparticles to emit UV and visible light upon NIR laser irradiation, driving the photoisomerization of azobenzene derivatives. This process triggered cyclic DNA hybridization and dehybridization, enabling the efficient release of doxorubicin in cancer cells. H. Zhan et al. [212] developed gold nanorods conjugated porous silicon nanoparticles encapsulated in alginate nanogels for the release of hydrophobic and hydrophilic therapeutic agents. The co-loading of therapeutics into functionalized nanogels significantly enhanced the inhibition of multidrug resistance through synergistic effects and accelerated cancer cell death when combined with photothermal therapy. Additionally, gold nanorods facilitated the conversion of NIR laser radiation into heat, which not only increased the release of the therapeutics but also induced thermal damage to cells, further promoting rapid cancer cell death.

#### 6.3.5. Magnetic-Responsive Release

Similarly to macro- or microgels, nanogels incorporate magnetic nanoparticles, such as Fe_3_O_4_, that respond to external magnetic fields to trigger controlled drug release. Soleimani et al. [213] developed a bioreducible and pH-responsive magnetic nanogel based on β-cyclodextrin, poly(2-ethyl-2-oxazoline,) and Fe_3_O_4_ nanoparticles for chemo/hyperthermia therapy, demonstrating significant doxorubicin loading efficiency and controlled release under magnetic and reductive conditions. Similarly, Massoumi et al. [214] synthesized a Fe_3_O_4_-loaded nanogel based on the copolymerization of *N*-isopropylacrylamide, maleic anhydride and starch. The system showed an optimized release of doxorubicin against both thermal and pH stimuli. These studies collectively underscore the versatility and efficacy of magnetic-responsive nanogels in advancing targeted drug delivery and combination therapies for cancer treatment. Another noteworthy application is in antimicrobial therapy. F. Gao et al. [215] developed multifunctional magnetic nanogels incorporating Fe_3_O_4_ nanoparticles and cationic poly(2-(dimethylamino)-ethyl methacrylate, demonstrating broad-spectrum antimicrobial activity against *Escherichia coli, Staphylococcus aureus*, and *Candida albicans*. This system presented special promise not only in tackling bacterial infection but also in stimuli-responsive applications such as protein adsorption and separation.

#### 6.3.6. Redox-Responsive Release

Similarly to macro- or micro-hydrogels, nanogels can be designed to be redox-responsive; therefore, they can respond to NADP^+^/NADPH, O_2_/$O_{2}^{-}$, or GSH-GSSG systems, among others, in higher concentrations in the intracellular environment. However, most of the redox-sensitive nanogels are designed to respond to GSH. Using this principle, Y. Wang et al. [216] discussed a redox-responsive system using disulfide bonds for GSH-triggered drug release. Polyethylene glycol was conjugated to poly(amidoamine) dendrimers via disulfide bonds, and cyclo(Arg-Gly-Asp-d-Phe-Cys) peptides were added to enhance targeting to integrin-overexpressed tumor cells. Doxorubicin release was triggered by elevated GSH levels within the tumor microenvironment. S. Wang et al. [218] prepared a lentinan-based nanogel using a disulfide-containing crosslinker to load diosgenin for chemo-immunotherapy. This nanogel inhibited the proliferation and metastasis of lung cancer cells. In addition, it promoted the proliferation of dendritic cells, increased the production of NO, and upregulated the expressions of the costimulatory molecules CD40, CD80, CD86, and MHC-II major histocompatibility complex class II due to the release of lentinan. Instead of including disulfide bond in the polymer chain, Tian et al. [217] developed diselenide-crosslinked zwitterionic nanogels with dual redox-labile properties under oxidative (H_2_O_2_) or reducing (GSH) conditions. These nanogels were prepared from 2-methacryloyloxyethyl phosphorylcholine and a diselenide bond-containing crosslinker and loaded with doxorubicin, showing a significant inhibitory effect against tumor cells.

#### 6.3.7. Enzyme-Responsive Release

Also, making use of the selectivity of enzymes against certain biomolecules, nanogels have been developed as enzyme-responsive systems. An example of enzyme-responsive nanogels based on the response to GOx was described by D. Zhou et al. [219], who prepared polymersomes from *N,N*-diethylaminoethyl methacrylate and 2-hydroxypropyl methacrylate incorporating GOx, as a glucose and pH dual responsive with multilevel self-regulation of blood glucose for insulin delivery. Using the capacity of GOx to convert glucose in glucuronic acid, the membrane permeability changed due to the protonation of amino groups at low pH, facilitating controlled insulin release. The system leverages pH sensitivity to regulate drug delivery in response to local acidic environments, such as inflammation or hyperglycemic conditions. As examples of MMT-sensitive systems, Massi et al. [220] described peptide-crosslinked nanogels that degraded in response to MMP-7, an enzyme overexpressed in inflammatory diseases and cancers. The nanogels were engineered for temperature-sensitive protein delivery. MMP-7 cleaved specific peptide sequences, enabling enzyme-triggered nanogel disassembly and the release of encapsulated therapeutic proteins. The nanogels were successfully loaded with model proteins and released their cargo in response to MMP-7 activity. Sensitive to MMP-9, Gordon et al. [221] designed derivatized (meth)acrylate-based nanogels, where MMP-9 cleaved specific peptide linkages, converting the surface of the nanogels from inactive to active properties to improve cellular uptake. This surface conversion enhanced their targeting ability in tumor microenvironments rich in MMP-9. These nanogels exhibited superior responsiveness to MMP-9, promoting greater uptake into cancer cells. Wu et al. [222] developed ALP-sensitive nanogels based on alginate-polyethyleneimine copolymers. The system successfully encapsulated and released doxorubicin in response to enzymatic triggers, where the nanogels were efficiently internalized by cells, demonstrated biocompatibility, and reduced cytotoxic effects compared to conventional carriers. Based on the action of hyaluronidases, J. Zhu et al. [223] developed a hybrid nanogel of aminoethyl methacrylate hyaluronic acid and methacrylated methoxy polyethylene glycol loaded with chlorhexidine for rapid hemostasis and antibacterial action, for wound healing. This nanogel presented low cytotoxicity and a prolonged release period of chlorhexidine, as well as rapid hemostasis capacity and accelerated wound healing in vivo using a mouse model.

## 7. Examples of FDA-Approved Hydrogels and Nanogels

As previously discussed, hydrogels and nanogels are extensively utilized for drug delivery, wound healing, and cosmetic and therapeutic interventions, with several formulations approved by regulatory agencies such as the FDA, underscoring their clinical relevance (Table 6). In drug delivery applications, hydrogels such as Cervidil^®^ and Zuplenz^®^ exemplify the versatility of these systems. Cervidil^®^, composed of a cross-linked hydrogel copolymer made from hexanetriol, macrogol 8000, and isocyanate, facilitates cervical ripening in pregnant women. This hydrogel provides localized and controlled release of dinoprostone, minimizing systemic side effects while mimicking natural tissue environments. Zuplenz^®^, comprising polyvinyl alcohol, macrogol 1000, and rice starch, is designed for managing nausea and vomiting induced by chemotherapy, radiation, or postoperative procedures. Its advantage lies in its patient-friendly, non-invasive oral delivery route. Hydrogels also play a pivotal role in wound management. 3M Tegaderm^®^, containing propylene glycol, is employed for low to moderate draining wounds, while DermaGauze^®^, an acrylate polymer hydrogel, addresses acute and chronic partial-thickness wounds. These formulations maintain a moist wound environment conducive to re-epithelialization and enhanced healing. However, they may be less effective for heavily exuding wounds and can require frequent replacement, potentially increasing patient discomfort and cost. Injectable hydrogels, such as Teosyal^®^, Belotero^®^, and Infuse^®^, highlight the adaptability of hydrogel systems in cosmetic and regenerative medicine. Teosyal^®^ and Belotero^®^, both based on hyaluronic acid (Belotero^®^ also includes lidocaine for pain reduction), are widely used for correcting facial wrinkles and folds. Their biocompatibility, immediate cosmetic results, and reversibility are significant advantages. However, these benefits are offset by risks such as local injection site reactions and the need for repeated treatments due to biodegradation. Infuse^®^, composed of collagen and recombinant human bone morphogenetic protein-2, is designed for use in spinal, oral, maxillofacial, and orthopedic surgeries. It effectively promotes bone regeneration, although complications such as ectopic bone formation and inflammatory responses have been reported. Regarding nanogels, Copaxone^®^, a nanogel composed of *l*-glutamate, *l*-alanine, *l*-lysine, and *l*-tyrosine random copolymers, is used to treat multiple sclerosis, reducing relapse rates and improving outcomes. However, its immunogenic potential and subcutaneous administration can result in local injection site reactions. Zilretta^®^, formulated with poly(lactic-co-glycolic acid) microspheres, delivers extended pain relief in osteoarthritis of the knee, reducing dosing frequency and improving patient adherence. Renagel^®^, a nanogel prepared from poly(allylamine hydrochloride), is used to manage hyperphosphatemia in chronic kidney disease by increasing circulation and therapeutic delivery. While effective, its gastrointestinal side effects and the high pill burden can pose adherence challenges.

## 8. Challenges, Advanced Applications, and Future Strategies

The development and application of advanced DDS have significantly transformed the area of Biomedicine in the last two decades, since more efficient, controlled, and targeted therapies for a variety of diseases have been carefully designed. Among the most promising materials, hydrogels and nanogels stand out due to their distinctive physical and chemical characteristics, such as their high water content, biocompatibility, and the capacity to encapsulate a broad spectrum of therapeutic moieties. Unfortunately, critical challenges including scale-up production, regulatory approval processes, and the need for enhanced targeting and releasing mechanisms remain uncertain. Among the most promising future strategies to tackle these obstacles and to foster the use of hydrogels and nanogels for drug delivery applications with the aim of more personalized clinical treatment are the following groups:

Advancements in material design and synthesis including tailored properties: one of the most critical areas to focus on is the design and synthesis of hydrogels and nanogels with tailored properties. Traditional hydrogels, often composed of synthetic or natural polymers, offer a broad range of properties, but to fully harness their potential for drug delivery, there is a need for more versatile and responsive materials. Interestingly, developing new smart and stimuli-responsive hydrogels or nanogels that can undergo reversible changes in their structure in response to external stimuli such as pH or temperature can significantly enhance the effectiveness of the release. Alternatively, the combination of hydrogels and nanogels may promote the advantages of each. For instance, biopolymers can improve stability, mechanical strength, and drug-loading capacity, which could be increased by incorporating nanoparticles into hydrogel networks to enhance the mechanical properties and loading efficiency.

Advancements in the fabrication design: although some examples have been included in this literature review, not so many authors have explored the possibility of combining different hydrogels or nanogels to achieve a final global objective, not achievable by one single hydrogel. In addition, additive manufacturing, also known as 3D printing, offers the possibility of fabricating advanced hydrogels based on the manufacturing process advantages offered by this technology. In addition, the combination of 3D printing with stimuli-response materials paved the way for advanced additive manufacturing, called 4D printing.

Targeted drug delivery through surface functionalization: to mitigate undesired secondary effects and systemic toxicity due to the lack of specificity in targeting diseased tissues, the surface functionalization with targeting moieties appears as a promising strategy. In this sense, ligand-based targeting based on ligands that incorporate antibodies, peptides, or small molecules that specifically bind to receptors and are overexpressed on target cells can improve the precision of drug delivery. Also, as an alternative functionalization with nanoparticles such as gold, silver, or magnetic nanoparticles can further enhance targeting for antibacterial or prosthesis applications. Also, more biomaterials such as polyethylene glycol (PEG) to avoid recognition and clearance by the immune system will be essential to develop in the near future to promote bioavailability.

Advanced drug loading and release mechanisms: to match therapeutic needs the development of new polymer networks, including nanogels matrixes with high superficial areas, is convenient to achieve higher drug entrapment and stability. Also, systems that provide dual-stimuli-responsive systems that release the drug in stages are often needed in tumor tissues and usually are not so easy to design. The modulation of biodegradation to reach the target and ensure bioactivity could also be modulated on the basis of the chemical structure of the gels.

Scale-up and manufacturing considerations: A major challenge in hydrogel and nanogel production is the development of scalable methods that ensure high quality, stability, and reproducibility. The transition from laboratory-scale synthesis to industrial-scale manufacturing requires addressing several technical and economic barriers. One of the most pressing issues is the high cost associated with the production of these materials. This includes the expense of raw materials, many of which are specialized polymers or biopolymers, as well as the energy-intensive nature of certain preparation methods, such as chemical crosslinking or advanced techniques like microfluidics and 3D printing. Furthermore, specialized equipment needed for precision synthesis and quality assurance adds to the financial burden, particularly for small-scale manufacturers or academic spin-offs attempting to commercialize these technologies. To overcome these economic challenges, there is a pressing need to explore innovative approaches such as using cost-effective, biodegradable polymers, optimizing reaction conditions to reduce energy inputs, and adopting scalable green chemistry techniques that minimize resource consumption and waste generation. Another significant hurdle in scaling up hydrogel and nanogel production is the issue of batch-to-batch variability. Even minor deviations in the raw material quality, environmental conditions (such as temperature, pH, or humidity), or synthesis parameters (e.g., polymerization time and crosslinking efficiency) can result in inconsistencies in the physicochemical properties of the final product. Such variability can compromise therapeutic efficacy, biocompatibility, and stability, posing challenges for clinical applications and regulatory approval. For example, slight differences in polymer chain length, crosslinking density, or nanoparticle size distribution can significantly affect drug release profiles, bioavailability, and patient outcomes. To address these issues, advanced quality control and process optimization strategies are essential. Automation of production processes, coupled with real-time monitoring systems, can help ensure consistent control over critical synthesis parameters. Techniques such as inline spectroscopy, advanced chromatography, and machine learning algorithms for predictive quality analysis are emerging as valuable tools to identify and mitigate sources of variability. Additionally, robust standard operating procedures (SOPs) and adherence to good manufacturing practices (GMP) are critical for maintaining consistent quality across production batches. Moreover, regulatory requirements for hydrogels and nanogels intended for therapeutic use further emphasize the need for stringent quality control. The variability in production must be minimized not only to satisfy commercial scalability but also to meet the standards of regulatory bodies such as the FDA and EMA. These agencies often require extensive validation of reproducibility, biocompatibility, and long-term stability before approving materials for clinical use, making batch-to-batch consistency a critical aspect of successful commercialization. Future progress in addressing these challenges will likely involve integrating novel manufacturing technologies, such as continuous flow reactors, which offer enhanced control over reaction conditions and scalability. Additionally, leveraging modular microfluidic systems can enable precise particle size control and high-throughput production, thereby reducing variability and improving scalability. Combining these innovations with sustainable practices, such as recycling solvents and minimizing chemical waste, could further enhance the feasibility of hydrogel and nanogel manufacturing for widespread therapeutic applications.

Regulatory challenges and clinical translation: while hydrogels and nanogels are widely regarded as biocompatible, their potential toxicity, long-term biodegradability, and immune responses require careful consideration, particularly for injectable applications. Toxicity may arise from residual monomers, crosslinking agents, or degradation by-products, which could accumulate in tissues or induce local cytotoxic effects. To minimize such risks, the use of non-toxic, FDA-approved materials and thorough purification protocols during synthesis is essential. Long-term biodegradability is another critical factor, as incomplete degradation or the formation of insoluble fragments could lead to accumulation and adverse effects. Materials designed with tunable degradation profiles, such as those based on naturally derived polymers (e.g., hyaluronic acid, chitosan), can mitigate these concerns by ensuring safe metabolization and excretion pathways. Immune responses, such as inflammation or hypersensitivity reactions, may occur due to the recognition of hydrogels or nanogels as foreign materials. Strategies such as surface functionalization with immunomodulatory agents, careful control of particle size, and the avoidance of immunogenic materials are key to mitigating these risks. Future comprehensive in vivo studies could be of major interest to better understand the long-term effects and safety profiles of these materials, thereby ensuring their suitability for clinical applications. Therefore, for these systems to succeed in the future, the creation of standardized methods is required to test them in both the preclinical and clinical stages, and regulatory agencies will need to update their guidelines to address the specific challenges posed by these advanced delivery systems, such as their dynamic properties or biodegradability.

Personalized medicine and precision drug delivery: these entities can play a crucial role in this paradigm by allowing the delivery of customized therapies based on the patient’s disease profile, genetics, or even response to treatment. In the future, tailored drug release profiles may be designed considering patient needs, involving rapid release for acute conditions or controlled release for chronic diseases. Also, the incorporation of biosensors into gel systems may allow for real-time monitoring of drug release and therapeutic responses to align this information with real needs in a dynamic way.

The future of hydrogels and nanogels in DDS holds tremendous potential. To realize this potential, continued advancements in material design, targeted delivery, drug release control, and scalable manufacturing will be crucial. By integrating these strategies are integrated along with regulatory challenges and personalized treatments, hydrogels and nanogels could become cornerstone technologies in the next generation of personalized, efficient, and safe drug delivery platforms.

## Figures and Tables

**Table 1:** Summary of conventional hydrogels, including materials used for their synthesis and their application.

Materials	Applications	Ref.
Calcium-crosslinked nanocellulose	Tissue regeneration Wound dressing	[58]
Alginate, hyaluronic acid, chitosan		[59]
Carboxymethyl, oxidized hydroxyethyl starch, chitosan		[60]
Oxadiazole group-decorated quaternary ammonium salts-conjugated poly(ε-caprolactone)-PEG-poly(ε-caprolactone)		[61]
Trans-1,4-cyclohexanediamine cross-linked with 1,3-dibromo-2-propanol		[62]
Alginate, chitosan, tetracycline in gelatine microspheres	Wound infection prevention	[63]
Bacterial peptides		[64]
Acrylic acid, *N,N′*-methylene bisacrylamide, silver, graphene		[65]
Bacterial cellulose, polyvinyl alcohol, silver nanoparticles,		[66]
Alginate, chitosan, ZnO		[67]
Alginate, CaCo_3_, growth factors	Healing promoting: Antioxidant and anti-inflammatory activity,	[68]
Chitosan, silk fibroin, L-proline		[69]
Carboxymethyl chitosan, epigallocatechin-3-O-gallate		[70]
Hydrophobically modified chitosan and oxidized dextrane	Direct wound treatment (Injectable hydrogels)	[72]
Glycol chitosan, dibenzaldehyde-terminated polyethylene glycol		[73]
Benzyl 3β-amino-11-oxo-olean-12-en-30-oate		[74]
Thiolated polyethylene glycol crosslinked with silver ions		[75]
Carboxymethyl chitosan, oxidized hyaluronic acid, blueberry anthocyanins		[76]
Collagen, chitosan, hyaluronic acid, poly(lactic-co-glycolic acid) microspheres with kartogenin. Collagen, chitosan, silk fibroin, polylysine-heparin sodium nanoparticles with TGF-β1.	Multi-approach wound management (Collagenous, osseous and epithelial tissue)	[77]
Alginate, polyvinyl alcohol, nanohydroxyapatite, chondroitin sulfate	Multi-approach wound management (Collagenous, osseous and epithelial tissue)	[78]
*N*-acryloyl 2-glycine, methacrylated gelatin		[79]
Methacrylated gelatin, *N*-acryloyl 2-glycine as crosslinker, M^2+^ ions, bioactive glass	Fabrication of complex structures	[80]
Polyacrylamide–polyacrylic acid crosslinked with Fe^3+^		[81]
Agarose, laponite nanosilicates	3D Bioprinting	[83]
Gelatin, fibrin, human umbilical vein endothelial cells, human neonatal dermal fibroblasts, and human bone marrow-derived mesenchymal stem cells		[84]

**Table 2:** Summary of pH-, temperature- and photo-sensitive hydrogels, including materials used for their synthesis and their application.

Scheme	Materials	Applications	Ref.
pH-Responsive	Carboxylated agarose, zinc ions, tannic acid	Healing capabilities Tissue regeneration	[89]
	Hydroxypropyl chitin, ferric ions, tannic acid		[90]
	Alginate, carboxymethyl cellulose		[91]
	Aldehyde-functionalized polymer, amine-modified silica nanoparticles	Cancer therapy	[96]
	*N*-carboxyethyl chitosan, PEG aldehyde		[97]
	Dextran phosphate-based hydrogel, prospidine		[98]
	Peptide hydrogel, paclitaxel		[99]
	OE polypeptides		[100]
	Methoxy PEG, cinnamaldehyde, adipic acid dihydrazide		[101]
	PEG, polyacrylic acid, ezetimibe	Hypercholesterolemia control	[102]
	Polycaprolactone, methacrylic acid copolymer	Radioprotective drug delivery	[103]
	*N*-Succinyl hydroxybutyl chitosan	Oral drug administration	[104]
	Chitosan nanospheres	Type 2 Diabetes management	[105]
Temperature- Responsive	PEG-poly(ε-caprolactone)-PEG	Hydrophobic drugs delivery	[106]
	Poly(ε-caprolactone-co-lactide)-β-PEG-β-poly(ε-caprolactone-co-lactide)	Protein drug delivery	[107]
	Methylcellulose, alginate		[108]
	Poly(ether urethane) with pH-sensitive mesoporous silica nanoparticles	Localized therapies	[109]
	PEG, *N*-(2-hydroxypropyl) methacrylamide-mono dilactate, thiolated hyaluronic acid	Neurological disorders treatment	[110]
	Chitosan-based hydrogel, triptolide-loaded nanostructures	Breast cancer therapy	[111]
	Chitosan, agarose		[112]
	Chitosan, β-glycerin sodium phosphate, glycyrrhetinic acid-modified graphene oxide	Hepatocellular carcinoma therapy	[113]
	Poloxamer, 5-fluorouracil	Colorectal cancer	[114]
Photo-Responsive	Chitosan, silk sericin Tegafur, Protoporphyrin IX	Chemotherapy and Photodynamic therapy	[115]
	Ti3C2Tx-based MXene		[116]
	Agarose, manganese oxide nanoparticles, Chlorin E6		[117]
	Silk fibroin		[118]
	4-Arm-PEG-SH, tannic acid, Fe^3+^ complex		[119]
	Chitosan, poly{2,5-thiophen-co-[3,6-di(thiophen-2-yl)-2,5-bis(*N,N,N*-trimethylhexan-1-aminiu-m)pyrrolo[3,4-c]pyrrole-1,4(2H,5H)-dione]-bromide-co-4,7-(2,1,3-benzothia-diazole)}	Antibacterial wound therapy	[120]
	PEG diacrylate, polyoxometalate, 2,2′-azobis[2-(2-imidazolin-2-yl) propane] dihydro-chloride		[121]
	Chitosan, CaO_2_ nanoparticles, MnO_2_ nanosheets		[122]
	Catechol functionalized chitosan, MnO_2_ nanosheets		[123]
	Agarose, tannic acid-Fe nanoparticles		[124]
	Gelatin, Cu^2+^ nanoparticles		[125]
	Hyaluronic acid, Fe^3+^-EDTA complexes		[126]

**Table 3:** Summary of magnetically- and redox-sensitive hydrogels, including materials used for their synthesis and their application.

Stimuli	Materials	Applications	Ref.
Magnetically Responsive	Dextran, Fe_3_O_4_ nanoparticles	Improving doxorubicin treatment (Cancer therapy)	[135]
	Silk fibroin, Fe_3_O_4_ nanoparticles		[136]
	Gelatin, alginate, Fe_3_O_4_ nanoparticles		[137]
	Poly(*N*-isopropylacrylamide), alginate, graphene oxide Fe_3_O_4_ nanoparticles		[138]
	Dopamine-conjugated hyaluronac crosslinked with Fe_3_O_4_ nanoparticles		[139]
	Tragacanth gum, acrylic acid Fe_3_O_4_ nanoparticles		[140]
	Tragacanth gum, *N*-isopropylacrylamide, 3-(trimethoxysilyl) propylmethacrylate, Fe_3_O_4_ nanoparticles	Improving methotrexate treatment (Cancer therapy)	[141]
	Carboxymethyl cellulose, β-cyclodextrin, chitosan, Fe_3_O_4_ nanoparticles		[142]
	Polyvinyl alcohol, cobalt ferrite nanoparticles	Wound healing therapy	[143]
	Poly(*N*-isopropyl acrylamide), alginate, MXene-wrapped Fe_3_O_4_@SiO_2_ nanoparticles		[144]
	Chitosan, cellulose, Fe_3_O_4_ nanoparticles		[145]
	Starch, itaconic acid, Fe_3_O_4_ nanoparticles, guaifenesin		[146]
	Alginate, polycaprolactone, Fe_3_O_4_ nanoparticles	Neurological disorders management	[148]
	Poly(lactic acid-ethylene glycol-lactic acid), Fe_3_O_4_ nanocubes		[149]
	Chitosan, β-glycerophosphate, Fe_3_O_4_ nanoparticles	Myocardial infarction management	[150]
	Carboxymethyl cellulose, acrylamide, *N,N′*-methylene bis acrylamide, Fe_3_O_4_-containing montmorillonite, diclofenac	Colon drug delivery	[151]
	Gelatin, liposomes loaded with ferulic acid, Fe_3_O_4_ nanoparticles	Cell development and faster tissue regeneration	[152]
Redox-Responsive	PEG-based polymers, pyridyl disulfide	Bovine serum albumin delivery	[153]
	Bis(2-methacryloyloxyethyl) disulfide, PEG, 2-(diisopropylamino) ethyl methacrylate	Tumor growth control Cancer Immunotherapy	[154]
	Calcium selenite/L-arginine nanospheres, Glucose oxidase, 6-aminonicotinamide, Sodium alginate		[155]
	Calcium phosphate, Polyacrylic acid, ROS-sensitive MnO_2_		[156]
	ε-Polylysine, pluronic F127, MnO_2_ nanosheets	Wond healing in diabetes	[157]
	Graphene derivates, cerium oxide nanoparticles	Neural stem cell regeneration	[158]

**Table 4:** Summary of enzyme-sensitive hydrogels, including materials used for their synthesis, the enzymes to which they are sensitive and their application.

Stimuli	Materials	Enzime Sensitivity	Applications	Ref.
Enzyme-Triggered	*N,N*-Diethylaminoethyl methacrylate, 2-hydroxypropyl methacrylate, GOx	GOx	Insulin release and diabetes management Diabetes management	[163]
	Cu_2_O/Pt nanocubes, alginate, hyaluronic acid, GOx			[164]
	Phenylboronic acids, diols, GOx			[165]
	Modified Concanavalin A, pullulan, GOx			[167]
	Carboxymethyl chitosan, polycaprolactone, MMP-2	MMP	Tissue regeneration	[168]
	Tetra-PEG-based hydrogel, carbon dots coupled with interleukin-4 plasmid DNA		Cardiac repair	[169]
	Methacryloyl sulfonated azocalix[4]arene, methacrylated hyaluronic acid		Osteoarthritis treatment	[170]
	Phosphorylated polyester, minocycline hydrochloride.	ALP	Periodontal treatment	[175]
	Phosphorylated peptide, paclitaxel		Cancer treatment	[176]
	Hyaluronic acid, cyclodextrin, doxorubicin	Hyaluronidase	Colorectal carcinoma treatment	[177]
	Chitosan, pectin, cisplatin. Sensitive to hyaluronidases		Cancer therapy	[178]
	Gelatin, *N*-(2-aminoethyl)-4-(4-(hydroxymethyl)-2-methoxy-5-nitrosophenoxy) butanamide, hyaluronic acid. Sensitive to hyaluronidases		Wound healing	[179]
	Histamine-modified hyaluronic acid crosslinked with Zr^4+^. Sensitive to hyaluronidases		Inhibition bacterial growth and biofilm formation	[180]

**Table 5:** Summary of nanogels divided according to their release mechanism, including materials used for their synthesis and their application.

Stimuli	Material	Applications	Ref.
Diffusion Controlled	Polyethylene glycol, 2,2-bis(hydroxymethyl)propionic	Chemotherapy	[200]
pH- Responsive	Poly(2-aminoethyl methacrylate hydrochloride) modified with 2,3-dimethylmaleic anhydride	Improving Doxorubicin Treatment	[201]
	*N*-Lysinal-N′-succinyl chitosan, poly(*N*-isopropylacrylamide), bovine serum albumin		[202]
	Chitosan-*graft*-poly(*N*-isopropylacrylamide) polymer, galactose		[203]
	Hydroxypropyl-β-cyclodextrin acrylate, chitosan derivatives	Antineoplasic treatment and Immunotherapy	[204]
	Methacrylic acid, *N*,*N*′-methylenebisacrylamide, camptothecin		[205]
	Chitosan, mPEG2000-isopropylideneglycerol		[206]
Thermo-Responsive	PNIPAM, Etanercept	Psoriasis management	[207]
	PNIPAM, vinyl-modified silica, doxorubicin	Improving Doxorubicin Treatment	[208]
	PNIPAM-polyglycerol	Genetin skin diseases treatment	[209]
Photo-Responsive	Polyacrylic acid derivatized with spiropyran components; gold nanoparticles/doxorubicin	Cancer therapy	[210]
	Polyacrylic acid, DNA-azobenzene, doxorubicin		[211]
	Alginate, silicon nanoparticles		[212]
Magnetic-Responsive	β-cyclodextrin, poly(2-ethyl-2-oxazoline), Fe_3_O_4_ nanoparticles	Cancer therapy	[213]
	*N*-Isopropylacrylamide, maleic anhydride, starch, Fe_3_O_4_ nanoparticles		[214]
	Poly(2-(dimethylamino)-ethyl methacrylate, Fe_3_O_4_ nanoparticles	Infections treatment	[215]
Redox-Responsive	Polyethylene glycol, poly(amidoamine) dendrimers, cyclo(Arg-Gly-Asp-d-Phe-Cys) peptides, doxorubicin	Improving Doxorubicin Treatment	[216]
	2-Methacryloyloxyethyl phosphorylcholine, doxorubicin		[217]
	Lentinan, Diosgenin	Chemo-immunotherapy	[218]
Enzyme-Responsive	*N,N*-Diethylaminoethyl methacrylate, 2-hydroxypropyl methacrylate	Diabetes management	[219]
	Peptide-crosslinking, therapeutic proteins	Inflammatory diseases treatment	[220]
	Derivatized (meth)acrylate, peptide linkages	Cancer therapy	[221]
	Alginate-polyethyleneimine copolymers		[222]
	Aminoethyl methacrylate hyaluronic acid, methacrylated methoxy polyethylene glycol, chlorhexidine	Wound healing	[223]

**Table 6:** Hydrogels and nanogels already approved by the FDA.

Category	Name	Composition	Approved Indication	Year of Approval	Reference
Hydrogel for drug delivery	Cervidil^®^	Hexanetriol, macrogol 8000, isocyanate cross-linked hydrogel copolymer	Initiation and/or continuation of cervical ripening in pregnant women at or near term	1993	[227]
Hydrogel for drug delivery	Zuplenz^®^	Polyvinyl alcohol, macrogol 1000, rice starch	Chemotherapy, radiation, and postoperative-induced nausea and vomitin	1991	[228]
Hydrogels for wound dressing	3M Tegaderm^®^	Propylene glycol	Low to moderate draining wounds, partial and full-thickness dermal ulcers	2018	[229]
Hydrogels for wound dressing	DermaGauze^®^	Acrylate polymer	Acute or chronic partial and full thickness wounds	2014	[230]
Injectable hydrogels	Teosyal^®^	Hyaluronic acid	Filling of facial wrinkles and folds	2017	[230]
Injectable hydrogels	Belotero^®^	Hyaluronic acid with lidocaine	Moderate to severe facial wrinkles and folds	2019	[230]
Injectable hydrogels	Infuse^®^	Collagen and recombinant human bone morphogenetic protein-2	Spine, oral, maxillofacial and orthopedic trauma surgeries	2002	[231]
Nanogel for drug delivery	Copaxone^®^	*l*-Glutamate, *l*-alanine, *l*-lysine, *l*-tyrosine random copolymer	Treatment of multiple sclerosis	1996	[232]
Nanogel for drug delivery	Zilretta^®^	Poly(lactic-*co*-glycolic acid) matrix microspheres	Extended pain relief over 12 weeks of osteoarthritis of the knee	2017	[233]
Nanogel for drug delivery	Renagel^®^	Poly(allylamine hydrochloride)	Increase circulation and therapeutic delivery in chronic kidney disease	2000	[234]
